# Coding Cell Identity of Human Skeletal Muscle Progenitor Cells Using Cell Surface Markers: Current Status and Remaining Challenges for Characterization and Isolation

**DOI:** 10.3389/fcell.2019.00284

**Published:** 2019-11-26

**Authors:** Sin-Ruow Tey, Samantha Robertson, Eileen Lynch, Masatoshi Suzuki

**Affiliations:** ^1^Department of Comparative Biosciences, University of Wisconsin, Madison, WI, United States; ^2^The Stem Cell and Regenerative Medicine Center, University of Wisconsin, Madison, WI, United States

**Keywords:** skeletal muscle, skeletal muscle progenitor cells, cell surface markers, human pluripotent stem cells, human induced pluripotent stem cells, muscular dystrophy, neuromuscular diseases

## Abstract

Skeletal muscle progenitor cells (SMPCs), also called myogenic progenitors, have been studied extensively in recent years because of their promising therapeutic potential to preserve and recover skeletal muscle mass and function in patients with cachexia, sarcopenia, and neuromuscular diseases. SMPCs can be utilized to investigate the mechanisms of natural and pathological myogenesis via *in vitro* modeling and *in vivo* experimentation. While various types of SMPCs are currently available from several sources, human pluripotent stem cells (PSCs) offer an efficient and cost-effective method to derive SMPCs. As human PSC-derived cells often display varying heterogeneity in cell types, cell enrichment using cell surface markers remains a critical step in current procedures to establish a pure population of SMPCs. Here we summarize the cell surface markers currently being used to detect human SMPCs, describing their potential application for characterizing, identifying and isolating human PSC-derived SMPCs. To date, several positive and negative markers have been used to enrich human SMPCs from differentiated PSCs by cell sorting. A careful analysis of current findings can broaden our understanding and reveal potential uses for these surface markers with SMPCs.

## Introduction

The most pronounced symptom of neuromuscular disorders is loss of skeletal muscle mass and strength, which causes functional decline and loss of independence in patients (Morrison, 2016; Mary et al., 2018). However, muscular deterioration is not always indicative of disease. Age-related progressive muscle atrophy and reduction of muscle function in healthy older adults is known as sarcopenia (Santilli et al., 2014; Ogawa et al., 2016; Marzetti et al., 2017). Although both a decrease in myocyte number (hypoplasia) and size (atrophy) are consistently observed in sarcopenic skeletal muscle (Deschenes, 2004; Narici and Maffulli, 2010), the precise biological mechanisms driving the precipitous decline in muscle mass and function are not well-understood. Current treatment options for these muscle conditions are only palliative, and to date there is no effective cure for any type of muscle wasting.

Stem cells offer the potential to become a realistic means to suppress the aging process in humans. To treat muscle wasting, stem cell-based therapy is the most attractive approach, as demonstrated in numerous pre-clinical studies and several clinical trials (Wilschut et al., 2012; Bajek et al., 2015; Ryder et al., 2017; Tompkins et al., 2017). For full muscle repair and regeneration, the formation of contractile muscle units is required. The obvious candidates for cell therapy are skeletal muscle progenitor cells (SMPCs, also known as myogenic progenitors). These cells can differentiate into skeletal myocytes with high efficiency and terminally achieve the formation of contractile muscle units, which is required for muscle repair and regeneration in the implanted muscle. There is a range of available cell sources to propagate SMPCs in culture, including from fetal muscle, adult muscle, and non-muscle somatic tissues. Furthermore, recent advances in stem cell technology allow us to use human pluripotent stem cells (PSCs) as a virtually indefinite new cell source for SMPC preparation. Human PSCs, which include embryonic stem cells (ESCs) and induced pluripotent stem cells (iPSCs), can overcome problems associated with expansion to large cell numbers for clinical use. Therefore, human PSC-derived SMPCs hold great promise for cell-based therapy to achieve muscle regeneration.

Not limited for use in cell-based regenerative therapy, human PSC-derived SMPCs are also available for *in vitro* modeling to study normal and pathological mechanisms in human skeletal muscle. As there is a large void between pre-clinical work carried out in rodent models and translating these therapies to humans, utilizing human PSC-derived SMPCs to study muscle wasting would help bridge the gap in knowledge. While *in vitro* culture systems have limitations and cannot completely recapitulate the complex *in vivo* milieu, they have powerful experimental advantages that enable us to study inaccessible human cell types in a controlled setting. Through *in vitro* drug screening using human PSC-derived SMPCs, we can possibly identify new mechanisms and molecules that have the capacity to prevent muscle wasting and atrophy during normal aging or disease processes.

This review catalogs the current findings on cell surface markers to identify human SMPCs. Here we focus on surface markers that have been reported in human PSC-derived SMPCs and compare their expression in other systems. Specific cell markers and/or cell surface proteins can be used for isolation, identification, and characterization of viable SMPCs. A better understanding of how SMPC markers are regulated *in vitro* and *in vivo* can help resolve enduring questions and challenges such as (1) the origins of SMPCs; (2) signaling mechanisms that drive lineage progression; (3) optimal isolation techniques; (4) selective enrichment of populations with clinical relevance, either for *in vitro* modeling and/or *ex vivo* therapy; and (5) potential genetic manipulations and/or pharmaceutical interventions to correct deteriorating muscle phenotypes. Similarities or differences in SMPC surface marker expression might be indicative of their stemness, myogenic differentiation propensity, and lineage potential to assume non-myogenic fates.

## Skeletal Muscle Development and SMPCs

There are various types of progenitor cells that have the ability to differentiate into skeletal myocytes. These cells include muscle satellite cells, muscle-derived stem cells (MDSCs), side population (SP) cells, mesoangioblasts and pericytes (reviewed in Hosoyama et al., 2014). Different sources have been used to propagate SMPCs in culture, including fetal muscle, adult muscle, non-muscle somatic tissues, and pluripotent stem cells (PSCs).

Skeletal muscle satellite cells are a type of adult SMPC localized beneath the basal lamina of adult muscle fibers. Regeneration of postnatal and adult muscles relies on satellite cells (Mauro, 1961; Starkey et al., 2011; Pallafacchina et al., 2013; Xu et al., 2015). These cells are mitotically quiescent in adult muscles. When the muscle is stimulated by stress or trauma, satellite cells are activated to divide, giving rise to daughter satellite cells to replenish the quiescent satellite cell pool and/or to undergo terminal differentiation for muscle repair (Bischoff and Heintz, 1994; Morgan and Partridge, 2003; Kuang et al., 2007; Le Grand et al., 2009; Xu et al., 2015). Both quiescent and activated satellite cells express Pax7 (Seale et al., 2000), whereas Myf5 is only expressed in activated satellite cells (Crist et al., 2012; Xu et al., 2015). With the expression of a muscle determinant factor MyoD, satellite cells are committed to become myoblasts, or myogenic precursor cells, which then terminally differentiate into multinucleated myotubes (Tapscott et al., 1988; Bischoff and Heintz, 1994; Seale et al., 2000; Morgan and Partridge, 2003; Kuang et al., 2007; Le Grand et al., 2009; Crist et al., 2012).

Muscle-derived stem cells (MDSCs) can be isolated from adult muscle biopsies by a combination of enzyme digestion and serial plating to collagen-coated culture plates, as these cells are less adhesive compared to other cell types in skeletal muscle (Vella et al., 2011). MDSCs are biologically, biochemically and genetically distinct from satellite cells (Qu-Petersen et al., 2002; Alessandri et al., 2004; Deasy et al., 2005; Usas et al., 2011). Human MDSCs are positive for CD105, CD133, vimentin and desmin, but negative for CD31, CD34, CD45, FLK-1/KDR, von Willebrand factor, VE-cadherins, and BCL2 (Alessandri et al., 2004). On the other hand, murine MDSCs have been known to express Sca-1 and CD34 (Cao et al., 2003; Deasy et al., 2005). Human MDSCs induced for myogenic differentiation in culture express striated-muscle actin, smooth-muscle actin, and desmin (Alessandri et al., 2004). Intramuscular transplant of human MDSCs was able to reverse muscle atrophy and promote phrenic nerve regeneration in pre-injured and immunocompetent alpha-sarcoglycan null mice (Lavasani et al., 2014). MDSCs can self-renew and differentiate into various cell types including non-muscle cells such as astrocytes, neurons, osteoblasts, chondrocytes, adipocytes, and cardiomyocytes (Deasy et al., 2001; Alessandri et al., 2004; Usas et al., 2011; Tchao et al., 2013). As demonstrated in a mouse model of acute hindlimb injury, multipotent MDSCs can be especially useful when muscle degeneration affects the myotendinous junction and the muscle-associated tendon. This is because MDSCs can potentially differentiate into fibroblasts or tenocytes to reconstitute the wasted muscle-tendon units (Hashimoto et al., 2016).

Side population (SP) cells are named after their ability to efflux the DNA-binding dye Hoechst 33342 and form a side population on Fluorescence Activated Cell Sorting (FACS) analysis (Goodell et al., 1996). This cell type was first identified in murine bone marrow as a subset of hematopoietic stem cells (HSCs; Goodell et al., 1996). Murine bone marrow SP cells were found to participate in muscle regeneration (Gussoni et al., 1999). Later, an isolation protocol was developed to enrich for murine skeletal muscle SP cells (Gussoni et al., 1999). These murine skeletal muscle SP cells were also able to replenish the satellite cell pool, which can contribute to muscle regeneration following systemic delivery (Gussoni et al., 1999; Asakura et al., 2002; Bachrach et al., 2006). While significant information is available for murine SP cells, studies in human skeletal muscle SP cells are relatively limited due to their scarcity, heterogeneity and difficulty to expand *in vitro*. It has been shown that human fetal muscle SP cells, which express CD146, Myf5, and Pax7, are highly myogenic and expandable *in vitro* but retain limited engraftment potential *in vivo* (Lapan et al., 2012).

Mesoangioblasts and pericytes are adult progenitor cells associated with vasculature running through the muscle tissue with a significant capacity to regenerate skeletal muscle. Mesoangioblasts are a type of mesodermal stem cell located in the walls of the embryonic aorta and the vessels of postnatal tissues (Tonlorenzi et al., 2007). As mesoangioblasts possess strong myogenic potential, systemic delivery of these cells into the blood stream enables them to reach and integrate into the target site of pathological muscles (Sampaolesi et al., 2003; Cossu et al., 2015). Intramuscular transplantation of muscle-derived CD133^+^ mesoangioblasts into a mouse cryoinjury model re-populated the satellite cell niche and was able to activate a regenerative response after subsequent re-injury (Meng et al., 2014). Interestingly, mesoangioblasts are known to express angiogenic cell markers such as CD34, Sca-1, and Fetal Liver Kinase 1 (De Angelis et al., 1999). Pericytes are embedded within the basement membrane of micro-vasculatures in adult skeletal muscles (Armulik et al., 2011; Cappellari and Cossu, 2013). These cells express Myf5 and Tissue-Nonspecific Alkaline Phosphatase (Dellavalle et al., 2007; Cappellari and Cossu, 2013).

A number of recent studies acknowledge that human PSCs, which include ESCs and iPSCs, can serve as a promising source for SMPC preparation. PSCs have the potential to differentiate into any cell lineage (Thomson, 1998; Takahashi et al., 2007). As iPSCs can be prepared using the somatic cells of a patient, they are a powerful tool for disease modeling, gene correction and drug screening in culture, and are a robust cell source for immuno-privileged clinical applications. In the last decade, SMPCs have been derived from PSCs via either transgene-based methods (i.e., overexpression of myogenic genes) or transgene-free approaches (i.e., supplementation of myogenic growth factors and/or signaling molecules in defined culture) (reviewed in Jiwlawat et al., 2018). Most protocols aim to generate satellite cell-like populations because satellite cells are considered *bona fide* skeletal muscle stem cells with both stem-like properties and myogenic activities. When deriving SMPCs from PSCs, especially via transgene-free approaches, the resulting cell populations commonly display high heterogeneity. Therefore, characterization and purification of PSC-derived SMPCs is crucial to promote culture expansion efficiency and increase engraftment rate following transplantation (Kim et al., 2017). Currently available methods for SMPC isolation often require fixation and intracellular staining, which prevents further examination of behavior and functionality after sorting. To preserve the viability of enriched transgene-free SMPCs, isolation should be based exclusively on unique surface markers that do not disrupt or compromise the integrity of the cells. Moreover, profiling by cell surface markers would also be valuable to identify how PSC-derived SMPCs resemble SMPC types such as mesoangioblasts, SP cells, and satellite cells.

## Cell Surface Markers to Define Human PSC-Derived SMPCs

Different molecular signatures are displayed in the SMPCs derived from various cell sources. Therefore, a profile of cell surface proteins can be used to define a specific cell type. A cell isolation procedure can use either positive selection or negative selection. Positive selection isolates the target cell type from the entire population, whereas negative selection depletes all other cell types of the population with only the target cells remaining. A combined use of different surface markers has worked successfully to enrich human PSC-derived SMPCs and deplete undesirable cell types by cell sorting. Although these individual works support the feasibility of SMPC sorting using multiple markers, such complicated procedures would critically dampen enthusiasm for potential therapeutic applications of PSC-derived SMPCs. In this section, we will introduce cell surface markers that can positively or negatively identify SMPCs derived from human ESCs and iPSCs (Table 1); however, this is not an exhaustive list. We will also summarize the expression of these markers on SMPCs from the other tissue sources. For consistency, SMPCs in this review are defined by: (1) ability to self-renew prior to differentiation induction and upon differentiation induction; (2) ability to form myofibers; and (3) expression of at least one protein marker that has been commonly seen in skeletal muscle progenitors, myoblasts, and myocytes. All cells and tissues mentioned onwards are sourced from humans, unless specified otherwise.

**Table 1:** Common markers to identify SMPCs from human PSCs and muscles.

		**PSCs**				**Adult muscle**		**Fetal muscle**		**Other sources (see NOTE)**
		**iPSCs**		**ESCs**						
		**Presence**	**References**	**Presence**	**References**	**Presence**	**References**	**Presence**	**References**	
Positive markers	CD10	**+**	Wu et al., 2018	**+**	Wu et al., 2018	**+**	Crisan et al., 2008; Lecourt et al., 2010; Wu et al., 2018	**+**	Crisan et al., 2008	
	CD13	**+**	Darabi et al., 2012; Tedesco et al., 2012	**+**	Darabi et al., 2012	**+**	Morosetti et al., 2006; Dellavalle et al., 2007; Crisan et al., 2008; Lecourt et al., 2010; Pisani et al., 2010b	**+**	Crisan et al., 2008; Castiglioni et al., 2014	
	CD29	**+**	Awaya et al., 2012; Darabi et al., 2012; Abujarour et al., 2014; Magli et al., 2017; Sakai-Takemura et al., 2018	**+**	Awaya et al., 2012; Darabi et al., 2012; Magli et al., 2017	**+**	Lecourt et al., 2010; Woodard et al., 2014; Charville et al., 2015; Xu et al., 2015; Garcia et al., 2018^*^; Lorant et al., 2018	**+**	Castiglioni et al., 2014	
	CD44	**+**	Darabi et al., 2012; Tedesco et al., 2012; Abujarour et al., 2014	**+**	Darabi et al., 2012	**+**	Morosetti et al., 2006; Dellavalle et al., 2007; Lecourt et al., 2010; Pisani et al., 2010b; Lorant et al., 2018	**+**	Crisan et al., 2008; Castiglioni et al., 2014	
	CD54	**+**	Magli et al., 2017	**+**	Magli et al., 2017					
	CD56	**+/–**	(+): Awaya et al., 2012; Darabi et al., 2012; Abujarour et al., 2014; Choi et al., 2016; Uezumi et al., 2016; Hicks et al., 2017; Sakai-Takemura et al., 2018 (–): Darabi et al., 2012; Awaya et al., 2012; Tedesco et al., 2012; Uezumi et al., 2016; Sakai-Takemura et al., 2018	**+/**low	(+): Barberi et al., 2007; Awaya et al., 2012; Darabi et al., 2012; Albini et al., 2013; Hwang et al., 2014, Goudenege et al., 2012; Hicks et al., 2017; Rao et al., 2018 (low): Caron et al., 2016	**+/–**	(+): Sinanan et al., 2004; Dellavalle et al., 2007; Zheng et al., 2007; Crisan et al., 2008; Lindström and Thornell, 2009; Negroni et al., 2009; Lecourt et al., 2010; Pisani et al., 2010a; Okada et al., 2012; Zheng et al., 2012; Agley et al., 2013; Bareja et al., 2014; Marg et al., 2014; Woodard et al., 2014; Xu et al., 2015; Alexander et al., 2016; Franzin et al., 2016; Uezumi et al., 2016; Garcia et al., 2018^*^; Lorant et al., 2018 (**–**): Proksch et al., 2009	**+**	Castiglioni et al., 2014; Hicks et al., 2017	Placenta (Park et al., 2011)
	CD63	**+**	Darabi et al., 2012	**+**	Darabi et al., 2012	**+**	Dellavalle et al., 2007			
	CD73	**+**	Awaya et al., 2012	**+**	Barberi et al., 2005, 2007; Awaya et al., 2012; Goudenege et al., 2012	**+**	Crisan et al., 2008; Lecourt et al., 2010; Woodard et al., 2014; Uezumi et al., 2016; Lorant et al., 2018			
	CD82	**+**	Uezumi et al., 2016; Sakai-Takemura et al., 2018			**+**	Alexander et al., 2016; Uezumi et al., 2016; Lorant et al., 2018	**+**	Alexander et al., 2016	
	CD90	**+**	Darabi et al., 2012	**+**	Darabi et al., 2012	**+/–**	(+): Morosetti et al., 2006; Dellavalle et al., 2007; Zheng et al., 2007; Crisan et al., 2008; Lecourt et al., 2010; Pisani et al., 2010b; Woodard et al., 2014; Uezumi et al., 2016; Lorant et al., 2018 (**–**): Proksch et al., 2009	**+**	Crisan et al., 2008; Castiglioni et al., 2014	
	CD105	**+/–**	(+): Awaya et al., 2012; Darabi et al., 2012 (–): Sakai-Takemura et al., 2018	**+**	Awaya et al., 2012; Darabi et al., 2012	**+/–**	Lecourt et al., 2010; Pisani et al., 2010b; Woodard et al., 2014; Uezumi et al., 2016; Lorant et al., 2018 (**–**): Alessandri et al., 2004	**+**	Crisan et al., 2008	Wharton's jelly (Conconi et al., 2006)
	CD146	**+/**low	(+): Darabi et al., 2012; Tedesco et al., 2012 (Low): Sakai-Takemura et al., 2018	**+**	Darabi et al., 2012	**+/**low	(+): Cerletti et al., 2006; Morosetti et al., 2006; Dellavalle et al., 2007; Crisan et al., 2008; Lecourt et al., 2010; Pisani et al., 2010b; Okada et al., 2012; Zheng et al., 2012 (Low): Lorant et al., 2018	**+**	Cerletti et al., 2006; Lapan et al., 2012; Alexander et al., 2016;	Placenta (Park et al., 2011)
	CD166	**+**	Awaya et al., 2012	**+**	Awaya et al., 2012; Darabi et al., 2012	**+**	Lecourt et al., 2010			
	CD184	**+/–**	(+): Borchin et al., 2013 (–): Awaya et al., 2012; Darabi et al., 2012	**+/–**	Awaya et al., 2012	**+**	Bareja et al., 2014; Marg et al., 2014; Garcia et al., 2018^*^	**+**	Castiglioni et al., 2014	
	CD271	**+**	Hicks et al., 2017; Sakai-Takemura et al., 2018	**+**	(+): Borchin et al., 2013 (**–**): Awaya et al., 2012; Darabi et al., 2012	**+**	Sakai-Takemura et al., 2018^*^	**+**	Alexander et al., 2016; Hicks et al., 2017	
	CD318	**+/–**	(+): Sakai-Takemura et al., 2018 (–): Uezumi et al., 2016			**+**	Uezumi et al., 2016; Lorant et al., 2018; Sakai-Takemura et al., 2018			
	CD362	**+**	Magli et al., 2017							
	ErbB3	**+**	Hicks et al., 2017; Sakai-Takemura et al., 2018	**+**	Hicks et al., 2017			**+**	Alexander et al., 2016; Hicks et al., 2017	
	c-Met	**+/–**	(+): Borchin et al., 2013 (**–**): Sakai-Takemura et al., 2018	**+**	Borchin et al., 2013					
	HLA-ABC	**+**	Darabi et al., 2012	**+**	Darabi et al., 2012					
	ITGA7	**+**	Darabi et al., 2012	**+**	Darabi et al., 2012	**+**	Castiglioni et al., 2014			
Negative marker	CD15					**–**	Lecourt et al., 2010; Pisani et al., 2010a; Agley et al., 2013			
	CD24	**–**	Wu et al., 2018	**–**		**–**	Wu et al., 2018			
	CD31	**–**	Darabi et al., 2012; Tedesco et al., 2012	**–**	Hicks et al., 2017	**–**	Alessandri et al., 2004; Dellavalle et al., 2007; Bareja et al., 2014; Charville et al., 2015; Xu et al., 2015; Garcia et al., 2018^*^; Lorant et al., 2018	**–**	Cerletti et al., 2006; Castiglioni et al., 2014	
	CD33	**–**	Darabi et al., 2012	**–**	Borchin et al., 2013					
	CD34	**–**	Awaya et al., 2012; Darabi et al., 2012	**+/–**	(+): Hwang et al., 2014 (**–**): Awaya et al., 2012; Darabi et al., 2012	**+/–**	(+): Proksch et al., 2009; Pisani et al., 2010a; Okada et al., 2012; Marg et al., 2014; Sidney et al., 2014 (**–**): Alessandri et al., 2004; Morosetti et al., 2006; Dellavalle et al., 2007; Zheng et al., 2007, 2012; Crisan et al., 2008; Negroni et al., 2009; Lecourt et al., 2010; Pisani et al., 2010b; Bareja et al., 2014; Charville et al., 2015; Franzin et al., 2016; Garcia et al., 2018^*^; Lorant et al., 2018	**–**	Crisan et al., 2008; Castiglioni et al., 2014	Placenta (Park et al., 2011) Umbilical cord blood (Koponen et al., 2007; Nunes et al., 2007)
	CD45	**–**	Darabi et al., 2012; Tedesco et al., 2012	**–**	Darabi et al., 2012	**–**	Alessandri et al., 2004; Morosetti et al., 2006; Dellavalle et al., 2007; Crisan et al., 2008; Proksch et al., 2009; Lecourt et al., 2010; Okada et al., 2012; Zheng et al., 2012; Bareja et al., 2014; Charville et al., 2015; Xu et al., 2015; Garcia et al., 2018^*^; Lorant et al., 2018	**–**	Crisan et al., 2008; Castiglioni et al., 2014, Franzin et al., 2016	Placenta (Park et al., 2011) Umbilical cord blood (Nunes et al., 2007)
	CD57	**–**	Borchin et al., 2013; Choi et al., 2016; Hicks et al., 2017; Sakai-Takemura et al., 2018	**–**	Darabi et al., 2012					
	CD106	**–**	Awaya et al., 2012; Darabi et al., 2012	**–**	Awaya et al., 2012; Darabi et al., 2012	**+/–**	(+): Sinanan et al., 2004; Lecourt et al., 2010 (**–**): Morosetti et al., 2006; Dellavalle et al., 2007; Crisan et al., 2008; Pisani et al., 2010b	**–**	Crisan et al., 2008	
	CD108	**–**	Sakai-Takemura et al., 2018			**+**	Sakai-Takemura et al., 2018			
	HLA-DR	**–**	Darabi et al., 2012	**–**	Darabi et al., 2012	**–**	Dellavalle et al., 2007			
	VEGFR2	**–**	Darabi et al., 2012; Tedesco et al., 2012	**–**	Darabi et al., 2012	**–**	Lorant et al., 2018			

*+, expression detected (>40% or not specified); low, expression detected but low (20–40%); –, expression not detected or very low (<20%). The Other sources column lists the sources where expression of “positive markers” was detected and expression of “negative markers” was not detected*.

* *Studies were done using postnatal muscle (Crisan et al., 2008); midterm placenta, fetal and adult muscle, fetal skin, fetal and adult pancreas, fetal bone marrow, fetal and adult adipose tissue (Franzin et al., 2016); fetal tissues, including skeletal muscle, myocardium, skin, pancreas, brain, bone marrow*.

### Positive Surface Markers for Human SMPCs

#### CD10

CD10 is commonly recognized as a cancer marker but is also used to identify specific populations of SMPCs (Maguer-Satta et al., 2011). This marker represents a type of endopeptidase protein also known as neprilysin, membrane metallo-endopeptidase, neutral endopeptidase, or common acute lymphoblastic leukemia antigen (Maguer-Satta et al., 2011). CD10 is also involved in hematopoiesis and B cell-innate immunology (Maguer-Satta et al., 2011). In a recent study using transgene-free PSCs and Pax7/Myf5 reporter ESCs, CD10^+^/CD24^−^ cells represented the SMPC population (Wu et al., 2018). However, CD10 expression does not always correspond to SMPCs. A study using adult human muscle showed that ~80% of Pax7^+^ cells expressed CD10, suggesting that not all adult satellite cells express CD10 (Wu et al., 2018). Human primary myoblasts also uniformly express CD10 (~97%) (Wu et al., 2018). When a CD146^+^/CD34^−^/CD45^−^/CD56^−^ cell subset was isolated from multiple human organs, co-expression of CD10 was identified in the isolated cells (Crisan et al., 2008).

#### CD13

CD13 is also known as aminopeptidase N, alanyl aminopeptidase or lamina-associated polypeptide 1. This marker was initially found to regulate adhesion and differentiation of muscle satellite cells following ischemic injury in mice (Rahman et al., 2014). CD13 plays important roles for the migration of cancer and endothelial cells (Kehlen et al., 2003; Fukasawa et al., 2006). In the human brain, CD13 is expressed on capillary pericytes (Smyth et al., 2018). Although CD13 has not been studied much in the context of human myogenic cells, one study reported that human adult pericytes were almost homogeneously positive (~97%) for CD13 (Dellavalle et al., 2007). Positive CD13 expression has also been detected in SMPCs derived from human PSCs (Darabi et al., 2012; Tedesco et al., 2012), adult muscle (Morosetti et al., 2006; Dellavalle et al., 2007; Lecourt et al., 2010; Pisani et al., 2010b), and fetal muscle (Castiglioni et al., 2014). Similar to CD10, co-expression of CD13 was observed in the CD146^+^/CD34^−^/CD45^−^/CD56^−^ cell subset isolated from multiple human organs (Crisan et al., 2008).

#### CD29

CD29, or integrin beta-1, is a well-established marker widely utilized to isolate or identify human myogenic cells. CD29 expression has been confirmed in PSC-derived SMPCs (Awaya et al., 2012; Darabi et al., 2012; Abujarour et al., 2014; Magli et al., 2017; Sakai-Takemura et al., 2018), fetal muscle (Castiglioni et al., 2014), postnatal muscle (Garcia et al., 2018), and adult muscle (Lecourt et al., 2010; Woodard et al., 2014; Charville et al., 2015; Xu et al., 2015; Lorant et al., 2018). An isoform of CD29, integrin beta-1D, has been shown to be severely reduced in patients with limb girdle muscular dystrophy type 2C (Anastasi et al., 2004) or sensitive-motor polyneuropathy (Anastasi et al., 2008). These results imply that normal levels of CD29 may be required for maintenance of healthy skeletal muscle.

In healthy adult skeletal muscle, all Pax7^+^ satellite cells displayed co-expression of CD56 and CD29, although a population of CD56^+^/CD29^+^ cells without Pax7 expression was still identified (Xu et al., 2015). Compared to an unselected cell population, CD56^+^/CD29^+^ cells exhibited a higher level of myofiber regeneration following cell transplantation into the pre-injured hindlimb muscle (tibialis anterior muscle) of immunodeficient NOD scid gamma (NSG) mice. In contrast, CD56^−^/CD29^+^ cells showed insufficient or non-existent myofiber regeneration (Xu et al., 2015). This study indicates that CD29 should be used in combination with other markers to define human SMPCs, as not only satellite cells but the myofiber and some non-muscle cells within skeletal muscle tissue also expressed CD29 (Xu et al., 2015). In a different study, when CD31^−^/CD34^−^/CD45^−^/CD29^+^/ epidermal growth factor receptor (EGFR)^+^ cells were isolated from human adult skeletal muscle, these cells expressed a series of myogenic cell markers such as Pax7, Pax3, Myf5, MyoD, Myogenin, and Myocyte Enhancer Factor 2C. Further, the isolated cells can differentiate into myotubes *in vitro* and when transplanted into NSG mice (Charville et al., 2015). In contrast, the remaining fraction (i.e., unselected cells) did not give rise to any Pax7^+^ cells and did not exhibit myogenic activity in culture, further supporting the notion that CD29 can indeed efficiently enrich myogenic cells when used in combination with other markers (Charville et al., 2015).

A study using an inducible gene expression system demonstrated that the expression of CD29/Integrin alpha-9 dimer (α9β1) was upregulated in human PSCs following Pax7 induction (Magli et al., 2017). In this study, Pax7-overexpressing cells were tagged with a green fluorescent protein (GFP) reporter protein using a Pax7 promotor. When α9β1^+^/CD54^+^ cells were isolated from Pax7-overexpressing PSCs, ~95% of cells were CD362-positive, and nearly 100% of GFP^+^ cells were triple positive (α9β1^+^/CD54^+^/CD362^+^). The triple positive cells demonstrated robust regenerative capacity *in vivo*, as human-specific satellite cells and myofibers were confirmed in the muscle of NSG mice at 10 months post-transplantation (Magli et al., 2017).

#### CD44

CD44 is also known as homing cell adhesion molecule, P-glycoprotein 1, lymphocyte homing receptor, extracellular matrix receptor III or HUTCH-1. The expression of CD44 is commonly seen in mesenchymal stem cells (MSCs) (Ramos et al., 2016), tenocytes (Scutt et al., 2008; Lui, 2015; Stolk et al., 2017), HSCs, and cancer stem cells (Thapa and Wilson, 2016; Senbanjo and Chellaiah, 2017). CD44 has also been detected on SMPCs from different cell sources, including PSCs (Darabi et al., 2012; Tedesco et al., 2012; Abujarour et al., 2014), adult muscle (Morosetti et al., 2006; Dellavalle et al., 2007; Lecourt et al., 2010; Pisani et al., 2010b; Lorant et al., 2018), and fetal muscle (Crisan et al., 2008; Castiglioni et al., 2014). Although CD44 expression in SMPCs was already verified in a number of previous works, this marker has not been tested to isolate or characterize human SMPCs by FACS. Interestingly, CD44 interacts with hyaluronan and activates EGFR signaling (Thapa and Wilson, 2016), which suggests that CD44 may play a significant role in the process of myogenesis through the EGFR-mediated pathway (Leroy et al., 2013). As an additional note, CD44 expression was identified in the endomysium of adult skeletal muscle (Lecourt et al., 2010).

#### CD54

CD54 (intercellular adhesion molecule 1, ICAM-1) is a glycoprotein typically expressed on endothelial cells and leukocytes (Zadeh et al., 2000; Long, 2011). CD54 expression is absent in healthy human muscle but detected in patients with inflammatory myopathies (Bartoccioni et al., 1994; De Bleecker and Engel, 1994; Marino et al., 2003). Treatment with various cytokines can induce CD54 expression in cultured human skeletal muscle cells (Goebels et al., 1992; Michaelis et al., 1993; Bartoccioni et al., 1994; Marino et al., 2001). One study reported that CD54 was upregulated by inducible Pax7 overexpression in human PSCs (Magli et al., 2017). Chromatin immunoprecipitation sequencing (ChIP-Seq) data revealed that Pax7 binds the 5′ region of the CD54 gene, suggesting that Pax7 directly regulates CD54 gene expression. Using the PSCs with inducible Pax 7 expression, CD54^+^/CD362^+^/integrin α9β1^+^ cells were able to repopulate the satellite cell pool and generate new muscle fibers in an NSG mouse model at 10 months post-transplantation. In the same study, the potential of CD54 as a sole marker was also evaluated for SMPC isolation. When using the PSCs with a GFP reporter gene driven by a Pax7 promotor, GFP^+^ cells were highly enriched by CD54-based selection. Notably, the isolated cells exhibited robust muscle differentiation *in vivo* without any signs of teratoma formation even after 12 months of transplantation in NSG mice (Magli et al., 2017).

#### CD56

CD56, or neural cell adhesion molecule (NCAM), has been utilized for isolation and identification of human myogenic cells in an extensive list of publications. CD56 expression is observed on SMPCs derived from PSCs (Barberi et al., 2007; Awaya et al., 2012; Darabi et al., 2012; Goudenege et al., 2012; Albini et al., 2013; Abujarour et al., 2014; Hwang et al., 2014; Caron et al., 2016; Choi et al., 2016; Uezumi et al., 2016; Hicks et al., 2017; Rao et al., 2018; Sakai-Takemura et al., 2018) postnatal muscle (Garcia et al., 2018), adult muscle (Sinanan et al., 2004; Dellavalle et al., 2007; Zheng et al., 2007, 2012; Crisan et al., 2008; Lindström and Thornell, 2009; Negroni et al., 2009; Proksch et al., 2009; Lecourt et al., 2010; Pisani et al., 2010a; Okada et al., 2012; Agley et al., 2013; Bareja et al., 2014; Marg et al., 2014; Woodard et al., 2014; Xu et al., 2015; Alexander et al., 2016; Franzin et al., 2016; Uezumi et al., 2016; Lorant et al., 2018), fetal muscle (Castiglioni et al., 2014; Hicks et al., 2017), and placenta (Park et al., 2011).

It has been deduced previously that CD56^+^/Pax7^−^ cells in adult muscle could represent activated satellite cells or myoblasts (Lindström and Thornell, 2009). Alongside other markers, CD56 expression has been used to enrich SMPCs, in which the isolated populations were able to form myotubes at high efficiency *in vitro* and/or contribute to muscle regeneration *in vivo* (Sinanan et al., 2004; Barberi et al., 2007; Dellavalle et al., 2007; Zheng et al., 2007, 2012; Crisan et al., 2008; Lecourt et al., 2010; Pisani et al., 2010a; Okada et al., 2012; Bareja et al., 2014; Castiglioni et al., 2014; Woodard et al., 2014; Xu et al., 2015; Alexander et al., 2016; Choi et al., 2016; Uezumi et al., 2016; Hicks et al., 2017; Lorant et al., 2018). CD56 expression was also used to identify and confirm SMPC fractions in the purified cells using other surface markers (Negroni et al., 2009; Proksch et al., 2009). Genetic analysis revealed that when compared to whole populations, both CD56^+^ cultured fetal myocytes and human PSC-derived SMPCs highly expressed genes associated with myogenesis, embryonic development and cell migration (Hicks et al., 2017).

However, several other studies indicate that CD56^−^ cells may also contain SMPCs. A population of CD146^+^/CD34^−^/CD45^−^/CD56^−^ cells were isolated from multiple human organs (Crisan et al., 2008) and placenta (Crisan et al., 2008; Park et al., 2011). The isolated cells, specifically named “perivascular cells” in these studies, were able to form multinucleated Myosin heavy chain (MyHC)^+^/desmin^+^/dystrophin^+^ myotubes *in vitro* as well as human spectrin^+^/human dystrophin^+^ myofibers and new blood vessels *in vivo*. After myogenic differentiation was induced in culture, these CD146^+^/CD34^−^/CD45^−^/CD56^−^ cells showed upregulated CD56 expression (Park et al., 2011). These cells were also able to differentiate into myotubes, chondrocytes, adipocytes and osteocytes upon appropriate inductions in culture (Crisan et al., 2008). In another report, CD146^+^/CD45^−^/CD56^−^/UEA-1R^−^ cells could generate a high number of human spectrin^+^ myofibers in an immunodeficient mouse model (Severe Combined Immunodeficiency, SCID; Zheng et al., 2012). Termed “endothelial cells” in the specific study, a CD45^−^/CD56^−^/CD34^+^/CD144^+^ population derived from adult muscle formed MyHC^+^ myotubes *in vitro* and a low number of human spectrin^+^ myofibers *in vivo* (Okada et al., 2012). More than half of these “endothelial cells” eventually expressed CD56 after expansion in culture (Okada et al., 2012).

The absence of CD56 expression may indicate diminished myogenic potential and increased adipogenic potential. In separate reports, CD56^−^/TE-7^+^, CD56^−^/CD15^+^, CD56^−^/CD34^+^, and CD56^−^/CD34^−^ cells derived from adult muscle were differentiated into adipoblasts but not myotubes (Zheng et al., 2007; Lecourt et al., 2010; Pisani et al., 2010a,b). When adult muscle-derived CD56^−^/CD29^+^ SMPCs were transplanted into NSG mice, human cell-derived myofibers were detected in only 1 out of 7 recipients, probably reflecting contamination with the CD56^+^/CD29^+^ fraction or a very limited myogenic potential of CD56^−^/CD29^+^ cells (Xu et al., 2015).

Based on these findings, CD56 expression seems to be identified in cells with a high capacity to differentiate into muscle, but if used alone may not work to enrich human SMPCs as a highly purified population.

#### CD73

Expression of CD73 (or ecto-5′-nucleotidase) has been detected in growth plates, articular cartilage, and hypertrophic chondrocytes (Coutu et al., 2017). CD73 is considered a marker of tenocytes (Lui, 2015; Stolk et al., 2017), MSCs (Ramos et al., 2016), vascular smooth muscle cells (Tamajusuku et al., 2006; Yang et al., 2015), and cancer cells (Gao et al., 2014). Although CD73 has not been frequently used for SMPC identification, its expression was found almost homogenously on SMPCs derived from several sources, including human PSCs (Awaya et al., 2012), MyoD-overexpressing human ESCs (Goudenege et al., 2012), and adult muscle (Woodard et al., 2014). These SMPCs expressed CD73, even though CD73 was not initially used for cell isolation (Crisan et al., 2008; Lecourt et al., 2010; Uezumi et al., 2016; Lorant et al., 2018). It has been reported that myogenic cells resided in the CD73^+^ fraction of ESC-derived MSCs, and that these cells were able to differentiate into myotubes, adipocytes, chondrocytes and osteoblasts *in vitro* (Barberi et al., 2005, 2007).

#### CD82

CD82, also known as Tespan-27, is a member of the tetraspanin protein family primarily identified as a metastasis suppressor (Tonoli and Barrett, 2005). Recently, CD82 has become a popular marker to use in identifying SMPCs, as this cell surface protein is expressed on SMPCs derived from human PSCs (Uezumi et al., 2016; Sakai-Takemura et al., 2018), adult muscle (Alexander et al., 2016; Uezumi et al., 2016; Lorant et al., 2018) and fetal muscle (Alexander et al., 2016). In healthy adult muscle, CD82 was detected on ~97% of Pax7^+^ or M-cadherin^+^ satellite cells and a small number of interstitial cells (Uezumi et al., 2016). CD82 expression is also maintained in activated and differentiating myogenic cells (Alexander et al., 2016). This marker can be used alone to enrich SMPCs with high regenerative capacity post-transplantation (Uezumi et al., 2016). When this marker was used alongside CD146 and CD56 on separate occasions, increased myogenic activity was observed in the CD82^+^ fractions both *in vitro* and *in vivo* (Alexander et al., 2016; Uezumi et al., 2016).

Recent studies support the idea that CD82 may play significant roles during the process of myogenesis. Overexpression of CD82 molecules enhanced muscle differentiation in primary myoblasts (Alexander et al., 2016), whereas its downregulation decreased their proliferation and differentiation (Alexander et al., 2016; Uezumi et al., 2016). In CD56^+^/CD82^+^ myogenic cells purified from human muscle, knockdown of CD82 increased the transcription of MyoD1 and Myogenin and led to premature differentiation even under culture conditions ideal for cell growth (Uezumi et al., 2016). Interestingly, a p38 inhibitor could suppress upregulation of MyoD1 and Myogenin induced by CD82 knockdown. These results implicate that CD82 likely regulates the balance between differentiation and self-renewal of SMPCs via a p38-mediated signaling pathway (Uezumi et al., 2016). CD82 forms a protein complex with integrin α7β1 and α-sarcoglycan, both of which have been linked to muscle disorders (Alexander et al., 2016). The muscles from patients with Duchenne muscular dystrophy (DMD) commonly showed a reduced expression of CD82, which suggests that this molecule may play essential roles of SMPC function in the process of muscle degeneration (Alexander et al., 2016).

#### CD90

CD90/Thy1 is a glycophosphatidylinositol-anchored glycoprotein frequently used as an MSC marker (Ramos et al., 2016). CD90 is also expressed on tenocytes (Scutt et al., 2008; Lui, 2015; Stolk et al., 2017), endothelial cells, HSCs, and in developing nervous tissues (Wetzel et al., 2004). A number of studies reported that CD90 expression was detected in SMPCs derived from human PSCs (Darabi et al., 2012), adult muscle (Morosetti et al., 2006; Dellavalle et al., 2007; Zheng et al., 2007; Proksch et al., 2009; Lecourt et al., 2010; Pisani et al., 2010b; Woodard et al., 2014; Uezumi et al., 2016; Lorant et al., 2018), and fetal muscle (Crisan et al., 2008; Castiglioni et al., 2014). In human adult muscle, CD90^+^ cells were also identified in the endomysium and adventitia of venous blood vessels (Lecourt et al., 2010). When culture-expanded satellite cells were sorted into CD34^+^/CD90^−^, CD34^−^/CD90^−^, and CD34^−^/CD90^+^ fractions, all three fractions expressed myogenic markers (Myf-5, MyoD, and Myogenin) as well as endothelial markers (CD31 and von Willebrand factor) (Proksch et al., 2009). Although CD90 expression was confirmed throughout various types of SMPCs, it remains inconclusive whether CD90 alone could represent the entirety of the SMPC population.

#### CD105

CD105 (endoglin, Src Homology 2) is a well-acknowledged marker of endothelial cells. This molecule plays a crucial role in angiogenesis and tumor growth (Fonsatti et al., 2010). Not limited to endothelial cells, the expression of CD105 is also identified in various types of SMPCs. This includes SMPCs derived from PSCs (Awaya et al., 2012; Darabi et al., 2012), adult muscle (Alessandri et al., 2004; Lecourt et al., 2010; Pisani et al., 2010b; Woodard et al., 2014; Uezumi et al., 2016; Lorant et al., 2018), and fetal muscle (Crisan et al., 2008), although the level of CD105 expression ranged widely from rare to ubiquitous in different types of progenitor cells. In adult muscle, CD105 was expressed in the sinusoidal endothelium and endomysium (Lecourt et al., 2010; Coutu et al., 2017). When CD105^+^/CD31^−^/KDR^−^ cells were isolated from Wharton's jelly (a gelatinous substance within the umbilical cord) and implanted into the muscle of Lewis male rats, the grafted cells were positive with sarcomeric tropomyosin, a protein that regulates muscle contraction, at 2 weeks post-transplantation (Conconi et al., 2006). These results indicate that the grafted CD105^+^ cells could generate functional muscle fibers. Further, these CD105^+^/CD31^−^/KDR^−^ cells were able to differentiate into myotubes, adipocytes and osteoblasts *in vitro*. In other studies, CD105 was also found on human tenocytes (Lui, 2015; Stolk et al., 2017). As such, CD105 may likely be indicative of mesodermal progenitor cells with multilineage potential.

#### CD146

CD146 (or melanoma cell adhesion molecule, MCAM) is well-studied for its important role in development, cell migration, immunology, angiogenesis, cancer progression, and myogenesis (Wang and Yan, 2013). In adult muscle, CD146 expression has been observed in the endomysium (Lecourt et al., 2010). In fetal skeletal muscle, CD146^+^ cells were found adjacent to myofibers and within blood vessels. These CD146^+^ cells were also positive for M-Cadherin, Pax7, and MyoD (Cerletti et al., 2006).

CD146 expression has been detected on SMPCs derived from PSCs (Darabi et al., 2012; Tedesco et al., 2012; Sakai-Takemura et al., 2018), adult muscle (Cerletti et al., 2006; Morosetti et al., 2006; Dellavalle et al., 2007; Crisan et al., 2008; Lecourt et al., 2010; Pisani et al., 2010b; Okada et al., 2012; Zheng et al., 2012; Lorant et al., 2018), fetal muscle (Cerletti et al., 2006; Crisan et al., 2008; Lapan et al., 2012; Alexander et al., 2016), and placenta (Park et al., 2011). It has been reported that the CD146^+^ fraction of SMPCs derived from adult muscle, fetal muscle and placenta contained cells with higher myogenic potential (Cerletti et al., 2006; Crisan et al., 2008; Park et al., 2011; Zheng et al., 2012; Alexander et al., 2016).

When both SP and main population (MP) cells were selected from human fetal muscle cells by scatter gating following FACS analysis, the CD146-based selection could enrich a population of myogenic cells. However, freshly isolated cells remained heterogenous: only ~50% of CD146^+^ SP and MP cells expressed Pax7, Myf5, or MyoD (Lapan et al., 2012). If SP and MP cells were sorted based on CD146 expression and then maintained in culture conditions that favored muscle differentiation, only CD146^+^ cell populations showed myotube formation. Interestingly, the highest number of myotube-positive cells was observed in the CD146^+^ SP fraction. Furthermore, CD146^−^ cell fractions subsequently acquired CD146 expression during expansion in culture but did not acquire myogenic potential (Lapan et al., 2012). These observations suggest that myogenic lineage enrichment based on CD146 may only be useful for freshly isolated cells. When transplanted into NOD mice, CD146^+^ SP cells exhibited high muscle regenerative capacity and gave rise to new human muscle fibers, whereas CD146^+^ total cells (i.e., a population containing both MP and SP cells) and CD146^+^ MP cells showed low engraftment rate (Lapan et al., 2012).

#### CD184

CD184, or commonly recognized as C-X-C chemokine receptor type 4 (CXCR-4), is a molecule with potent chemotactic activity for lymphocytes. It is crucial for homing hematopoietic stem cells to their adult marrow (Villa et al., 2012). CD184 is widely expressed on blood cells, endothelial cells, and neural cells (Walenkamp et al., 2017). CD184 expression is also confirmed on PSC-derived SMPCs (Borchin et al., 2013) as well as in adult muscle (Bareja et al., 2014; Marg et al., 2014; Garcia et al., 2018) and fetal muscle (Castiglioni et al., 2014).

As CD184 is also expressed in neural cells (Kos et al., 1999) and definitive endoderm cells (Teo et al., 2012), a combination of CD184 and other cell markers would be necessary to eliminate these non-myogenic cell types in PSC-derived cell populations. For instance, one study used the depletion of CD57^+^ cells and c-Met^−^ cells to sufficiently exclude neural cells among the CD184^+^ cell population (Borchin et al., 2013). In this study, CD57^−^/acetylcholine receptor (AChR)^−^ cells were purified from differentiated PSCs and then sorted into c-Met^+^/CD184^+^, c-Met^+^/CD184^−^, c-Met^−^/CD184^+^, and c-Met^−^/CD184^−^ fractions. Immunocytochemical analysis immediately after sorting revealed that both c-Met^+^/CD184^+^ and c-Met^+^/CD184^−^ fractions contained highly pure SMPCs. In contrast, c-Met^−^/CD184^+^ fraction contained both SMPCs and SOX1^+^ neural cells, and c-Met^−^/CD184^−^ fraction did not have any SMPCs. Although CD184 alone could not enrich SMPCs, CD184 expression may be indicative of myogenic lineage progression. Interestingly, both c-Met^+^/CD184^+^ and c-Met^+^/CD184^−^ fractions expressed early myogenic genes Six4 and Pax3 (paralogue of Pax7) at day 23 of post-myogenic differentiation of PSCs (Borchin et al., 2013). Both c-Met^+^/CD184^+^ and c-Met^+^/CD184^−^ cells gradually acquired Pax7 expression by day 25, and almost all these cells exhibited co-expression of Pax3 and Pax7 by day 35. At this point, the number of Pax7-positive cells was higher in c-Met^+^/CD184^+^ cells compared to c-Met^+^/CD184^−^ cells, while 97–98% of cells still retained Pax3 expression in both preparations. As Pax7 has been considered a later-stage marker of myogenic progenitors compared to Pax3, a specific population in c-Met^+^/CD184^+^ cells might have proceeded farther along the differentiation process. On the other hand, c-Met^+^/CD184^−^ cells might represent a more primitive SMPC population than c-Met^+^/CD184^+^ cells. Furthermore, gene-expression analysis confirmed the presence of Pax3 and Pax7 mRNA transcripts together with LBX1 in both c-Met^+^/CD184^+^ and c-Met^+^/CD184^−^ sorted populations. These *in vitro* results seem to be consistent with the situations observed during *in vivo* muscle development: within the hypaxial domain of the embryonic dermomyotome, the delamination of Pax3^+^/LBX1^+^ migratory muscle precursors is dependent on c-Met expression (Bladt et al., 1995; Dietrich et al., 1999), whereas CD184 is essential for the subsequent survival and distribution of precursors at the site of migration (Vasyutina et al., 2005; Buckingham, 2006).

#### CD271

CD271, also known as p75 neurotrophin receptor or low-affinity nerve growth factor receptor (p75NGFR), has recently been recognized as a positive marker to identify SMPCs and MSCs (Álvarez-Viejo et al., 2015; Sakai-Takemura et al., 2018). This surface marker was first reported as a candidate SMPC marker in a study when CD271 expression was detected in culture-expanded postnatal myoblasts but not in fibroblasts (Sakai-Takemura et al., 2018). When terminally differentiated, myotubes were exclusively formed in the CD271^+^ fraction of iPSC-derived SMPCs although some CD271^+^ cells remained non-myogenic (Sakai-Takemura et al., 2018). The progenitor cells sorted with a combination of CD271^+^ and other markers (CD57^−^/CD108^−^/ErbB3^+^) were able to generate myofibers with high efficiency *in vitro* and *in vivo* (Sakai-Takemura et al., 2018). The other group prepared CD271^+^ SMPCs from DMD patient-derived iPSCs (DMD iPSCs) that had been genetically corrected by CRISPR/Cas9 technology. These CD271^+^ SMPCs exhibited superior myotube-forming potential; even higher than the cells in the CD56^+^, CD146^+^, or CD184^+^ fractions (Hicks et al., 2017). In a different study, SMPCs derived from four human PSC lines (ESCs, wild type iPSCs, DMD iPSCs, and genetically corrected DMD iPSCs) commonly expressed CD271 and ErbB3. These CD271^+^/ErbB3^+^ cells displayed significantly higher expression of myogenic genes and increased efficiency of skeletal muscle differentiation (Hicks et al., 2017). When using genetically corrected DMD iPSC-derived CD271^+^ cells, the cells showed better engraftment in the muscle of dystrophin-deficient NSG mice compared to CD56^+^ cells (Hicks et al., 2017).

At early developmental stages before any other myogenic surface markers are presented on muscle progenitors, CD271/ErbB3-based isolation can be used to enrich Pax7^+^ cells and Myf5^+^ cells from human fetal muscles (Hicks et al., 2017). At 8 weeks of gestation, a CD271^+^/ErbB3^+^ subpopulation emerged in the muscle with expression of myogenic transcription factors. At 11 weeks, the CD271^+^/ErbB3^+^ subpopulation began to co-express some cell surface markers such as CD56, CD82, and CD146. These cells then started losing CD271 expression at 16 weeks of gestation. When CD271^+^/ErbB3^+^ cells were isolated at this time point, plated down, and differentiated in culture, the cells could form myotubes with nearly 100% efficiency in cell fusion (Hicks et al., 2017). Such changes of CD271 expression have been characterized during transitions from early to late waves of human fetal myogenesis, which also correlates to the development of primary limb myofibers or maturation of secondary fetal myofibers (Hicks et al., 2017). CD271/ErbB3 expression can be used to distinguish premature SMPCs from more committed MyoD^+^ myocytes (Hicks et al., 2017). Together, these data supports the idea that CD271 can be used as a positive selection marker to enrich human PSC-derived SMPCs.

However, we should bear in mind that CD271 expression may not always correlate to a capacity of muscle differentiation in human PSC-derived SMPCs. A recent study, which had used a double reporter ESC line driven by Pax7 and Myf5 promoter genes, revealed that CD271^+^/ErbB3^+^, CD271^+^/ErbB3^−^, and CD271^−^/ErbB3^+^ fractions contained similar proportions of Pax7^+^ and Myf5^+^ cells (Wu et al., 2018). When CD271^+^/ErbB3^+^, CD271^+^/ErbB3^−^, and CD271^−^/ErbB3^+^ fractions were sorted from Pax7/Myf5 dual reporter ESCs and terminally differentiated *in vitro*, all three fractions exhibited similar proportions of MyHC^+^ cells and displayed similar myotube formation efficiency (Wu et al., 2018).

#### ErbB3

As mentioned in the previous section, ErbB3 has also been used as a surface marker to efficiently purify human PSC-derived SMPCs (Hicks et al., 2017; Sakai-Takemura et al., 2018). ErbB3, also known as human epidermal growth factor receptor 3, is widely expressed in a variety of organs during human development, including skin, bone, muscle, nervous system, lungs, and intestines (Coussens et al., 1985). ErbB3 is also expressed in human fetal CD146^+^ cells which demonstrate high myogenic capacity (Alexander et al., 2016). When iPSC-derived SMPCs were sorted solely based on ErbB3 expression, myotubes were exclusively formed in the enriched ErbB3^+^ population following terminal differentiation in culture (Sakai-Takemura et al., 2018). ErbB3-based cell sorting could enrich myogenic cells much more efficiently compared to the isolation using a combination of CD56 and CD82 expression (Sakai-Takemura et al., 2018). In a different study, isolated iPSC-derived SMPCs with CD57^−^/CD108^−^/CD271^+^/ErbB3^+^ or CD271^+^/ErbB3^+^ were able to differentiate into myotubes with high efficiency in culture and *in vivo* (Sakai-Takemura et al., 2018). In a similar study using genetically corrected DMD iPSC-derived SMPCs, ErbB3^+^ cells displayed high potential for myotube formation compared to the cells solely sorted by CD56, CD146, CD184, or CD271 expression (Hicks et al., 2017). When ErbB3^+^ cells were prepared from four PSC lines (ESCs, wild type iPSCs, DMD iPSCs, and genetically corrected DMD iPSCs), all ErbB3^+^ cells from the four PSC lines were positive with CD271. These ErbB3^+^/CD271^+^ SMPC lines exhibited higher capacity for muscle differentiation than double-negative fractions (Hicks et al., 2017). When iPSC-derived SMPCs were transplanted into the limb muscle of dystrophin-deficient NSG mice, the implanted cells promoted significant engraftment and restored dystrophin expression in the grafted area (Hicks et al., 2017).

As we already introduced in the section on CD271, CD271/ErbB3 expression can be used to enrich Pax7^+^ and Myf5^+^ cells from fetal muscles at the first and second trimesters of human development (Alexander et al., 2016). A CD271^+^/ErbB3^+^ subpopulation began to emerge in fetal muscle at 8 weeks of gestation and gradually increased the expression of myogenic genes (Hicks et al., 2017). If CD271^+^/ErbB3^+^ cells were isolated at 16 weeks of gestation, the cells could differentiate into myotubes with a fusion efficiency close to 100% when plated down and differentiated in culture (Hicks et al., 2017). The changes in CD271^+^/ErbB3^+^ expression possibly mark the progression of human fetal myogenesis.

Similar to CD271, ErbB3 expression is not always indicative of higher myogenic potential in human PSC-derived SMPCs. In another study, ESC-derived cells were sorted to three fractions based on CD271 and ErbB3 expression (CD271^+^/ErbB3^+^, CD271^+^/ErbB3^−^, and CD271^−^/ErbB3^+^) and then myogenic differentiation was induced. Among the three fractions, there was no difference in the number of Pax7^+^ cells and Myf5^+^ cells (Wu et al., 2018). When ESC-derived Pax7/Myf5 double reporter cells were sorted into three fractions and the cells in these fractions were plated for myotube differentiation, neither the efficiency of myotube formation nor the percentage of MyHC^+^ cells were different when compared between three fractions (Wu et al., 2018).

#### CD318

CD318, also known as CUB domain-containing protein 1 (CDCP1) or transmembrane and associated with Src kinases (TRASK), is present on epithelial cells (Spassov et al., 2009), hematopoietic cells (Conze et al., 2003), MSCs (Buhring et al., 2004), and tumor cells (Uekita and Sakai, 2011). Some recent studies reported CD318 expression in myogenic cells. In satellite cells, cytoplasmic expression of CD318 was detected in ~75% of total cells (Uezumi et al., 2016). Pax7^+^/MyoD1^+^ adult muscle-derived SMPCs grown in hypoxic conditions were reportedly only found in the CD318^+^ fraction (Uezumi et al., 2016). In mice, satellite cells and myofibers positive with human cell specific markers were observed in recipients of CD318^+^ adult muscle-derived cells, but rarely in recipients of CD318^−^ cells (Uezumi et al., 2016). In contrast, it remains controversial whether CD318 can specifically identify SMPC pools in differentiated human iPSCs. To date, both the presence (Sakai-Takemura et al., 2018) and absence (Uezumi et al., 2016) of CD318 expression have been reported in two different studies. Such controversial results in CD318 expression may be caused by differences in culture conditions like oxygen concentration, as several studies demonstrated that CD318 expression was changed by hypoxia (Razorenova et al., 2011; Emerling et al., 2013; Cao et al., 2015). Since commercially available myoblasts and iPSC-derived cells cultured in normoxic conditions showed diminished or undetectable CD318 expression, CD318-based sorting failed to enrich myogenic cells in iPSC-derived SMPCs (Uezumi et al., 2016).

### Negative Surface Markers for Human SMPCs

The negative markers listed in Table 1 are typically absent on human SMPCs but mainly present on non-myogenic cell types. As the markers on their own are usually not indicative of SMPC properties, the cells positive with these markers should be depleted during SMPC isolation. In other words, a majority of SMPCs reside in the negative fraction of these markers. In this section, several representatives of negative surface markers are introduced.

#### CD15

CD15 (3-fucosyl-N-acetyl-lactosamine, Lewis X or stage-specific embryonic antigen 1) has been used to separate myogenic and adipogenic cells. CD15^+^ cells can be found outside adult muscle fibers (Lecourt et al., 2010); more specifically, in the interstitial position between the basal lamina of adjacent myofibers (Pisani et al., 2010a). Based on recent observations, CD15 seems to be negative in a majority of SMPCs because CD15 is predominantly detectable in the adipogenic cells with relatively less myogenic capacity. Cell sorting by CD56 and CD15 expression (CD56^+^/CD15^−^, CD56^−^/CD15^+^, and CD56^+^/CD15^+^) could distinguish three cell types: myogenic cells without adipogenic capabilities, non-myogenic cells with adipogenic capabilities, and myogenic cells with adipogenic capabilities, respectively (Lecourt et al., 2010; Pisani et al., 2010a; Agley et al., 2013). CD56^+^/CD15^+^ cells were still able to retain muscle differentiation capacity even under conditions that strongly favor adipogenesis (Agley et al., 2013).

#### CD24

Known as a sialoglycoprotein and a cell adhesion molecule, CD24 is commonly expressed on the cellular membrane of B lymphocytes and neutrophils (Elghetany and Patel, 2002; Tan et al., 2016), as well as on neural progenitors (Poncet et al., 1996; Fang et al., 2017; Gilliam et al., 2017). Depletion of CD24-expressing cells can enrich human PSC-derived SMPCs when used in combination with positive selection of CD10^+^ cells. When CD10^+^/CD24^−^ cells were enriched from a variety of PSC lines (Pax7/Myf5 double reporter ESCs, two reporter-free ESC lines, and one reporter-free iPSC line), the isolated cells showed a high level of myotube formation following terminal differentiation (Wu et al., 2018). CD24 expression was not detectable in primary myoblasts (Wu et al., 2018). Interestingly, specific deletion of Myogenin in adult SMPCs resulted in a 10-fold downregulation of CD24 (Meadows et al., 2008). At 16 and 19 weeks of human gestation, a specific cell population with strong CD24 expression was identified in the mesenchymal areas adjacent to developing muscles and intramuscular nerves (Figarella-Branger et al., 1993). In adult muscles, CD24 expression was identified in some unmyelinated nerve fibers and interstitial elongated cells near the neuromuscular junctions (Figarella-Branger et al., 1993). CD24-positive signals were also detected in regenerating muscle fibers following segmental necrosis (Figarella-Branger et al., 1993). These *in situ* locations of CD24 expression implicate that CD24 is possibly involved in the development, maintenance, and regeneration of muscle innervation. In mice, CD24 is expressed by myofiber synaptic nuclei and plays a role in synaptic transmission (Jevsek et al., 2006).

#### CD31

CD31, specifically named platelet endothelial cell adhesion molecule (PECAM-1), is a member of the immunoglobulin superfamily. CD31 is primarily used to detect endothelial cells (Lertkiatmongkol et al., 2016). For SMPC purification, this surface marker has been used as a negative selector. In a number of different studies, a depletion step of CD31^+^ cells has been applied to isolate SMPCs from adult muscle (Bareja et al., 2014; Xu et al., 2015; Garcia et al., 2018) and fetal muscle (Cerletti et al., 2006; Castiglioni et al., 2014). In adult muscle, CD31 expression was observed in the endomysium and intimae part of arteries (Lecourt et al., 2010). In a previous study using fetal muscle, both CD146^+^/CD31^+^ and CD146^+^/CD31^−^ cells were found adjacent to myofibers, and some CD146^+^/CD31^+^ were clearly located within blood vessels (Cerletti et al., 2006). When these cells were isolated from the tissues and cultured, both CD146^+^/CD31^+^ and CD146^+^/CD31^−^ cells were positive with myogenic markers such as Pax7, MyoD, and desmin. Notably, both cell types were able to differentiate into MyHC^+^ myotubes. These results suggest that CD31 expression itself would not correspond to the ability of muscle differentiation in isolated cells (Cerletti et al., 2006).

#### CD34

Although CD34 has been considered as a marker of satellite cells in mice, it does not mark satellite cells in human muscle (Péault et al., 2007). CD34 has been used in human muscle satellite cells for negative selection (Sidney et al., 2014). This surface protein has often been utilized for human SMPC isolation as a negative selection marker along with a combination with other surface markers (Zheng et al., 2007; Crisan et al., 2008; Proksch et al., 2009; Pisani et al., 2010b; Okada et al., 2012; Bareja et al., 2014; Castiglioni et al., 2014; Garcia et al., 2018). CD34 is a transmembrane phosphoglycoprotein and widely accepted as a marker of hematopoietic lineage (Sidney et al., 2014). However, CD34 expression is also detected on various types of non-hematopoietic cells such as MSCs (Huss, 2000; Lin et al., 2012), interstitial cells (Popescu et al., 2007; Rasmussen et al., 2007; Zheng et al., 2007; Yu et al., 2012), epithelial progenitors (Blanpain et al., 2004), vascular endothelial progenitors (Fina et al., 1990), and corneal keratocytes (Poole et al., 1993; Polisetty et al., 2008). Although several publications reported CD34 expression in muscle satellite cells (Sidney et al., 2014) and other myogenic cells (Zheng et al., 2007; Proksch et al., 2009; Pisani et al., 2010a; Okada et al., 2012), it remains controversial whether this marker itself is commonly detectable in different types of SMPCs from various sources. Nonexistent or low levels of CD34 expression were detected in human SMPCs derived from PSCs (Awaya et al., 2012; Darabi et al., 2012; Hwang et al., 2014), adult muscle (Alessandri et al., 2004; Morosetti et al., 2006; Dellavalle et al., 2007; Negroni et al., 2009; Lecourt et al., 2010; Zheng et al., 2012; Charville et al., 2015; Franzin et al., 2016; Lorant et al., 2018), fetal muscle (Castiglioni et al., 2014), umbilical cord blood (Koponen et al., 2007; Nunes et al., 2007), and placenta (Park et al., 2011). In skeletal muscle, CD34 expression represents an endothelial nature due to the detection of CD34^+^ cells in the endomysium and interstitial spaces (Hollemann et al., 2008; Lecourt et al., 2010; Pisani et al., 2010b). CD34 expression has also been detected in the blood vessels within skeletal muscle, particularly the tunica adventitia in veins and the tunica media in arteries (Lecourt et al., 2010).

Evidence in previous studies suggests that both CD34^+^ and CD34^−^ cells were present in SMPC pools, but the CD34^−^ cell population seemed to be more homogeneous and committed to the myogenic lineage compared to CD34^+^ cells. For instance, some groups of SMPCs have been confirmed as CD34^−^ populations, which include proliferative and activated satellite cells (CD34^−^/CD56^+^/Myf5^+^ cells), and a minority of pericytes and mesoangioblasts with adipogenic potential (CD34^−^/CD56^−^ cells) (Péault et al., 2007). In contrast, CD34^+^ cells might contain several cell populations originated from the interstitial compartment in skeletal muscle, such as myoendothelial cells, endothelial cells, and SP cells. Interstitial CD34^+^ cells may have been derived from resident endothelial cells (Vailhe et al., 2001) or have invaded skeletal muscle directly via circulation (Asahara and Kawamoto, 2004). Typically, CD34^−^ cells show consistent myogenic potential across different preparations of SMPCs, whereas myogenic potential in CD34^+^ cells tends to be inconsistent and shows variation in different preparations of SMPCs.

A series of studies using adult muscle-derived MDSCs also supports the efficiency of CD34-based isolation to enrich homogenous myogenic cells in the negative fraction. Regardless of muscle type and culture period, CD34^−^ MDSCs displayed high expression of myogenic markers (Myf5, Pax7, MyoD, myogenin, desmin, and muscle creatine kinase) (Pisani et al., 2010b). These CD34^−^ MDSCs consistently expressed CD56 and showed myogenic potential following differentiation (Pisani et al., 2010b). In contrast, CD34^+^ MDSCs barely expressed these myogenic markers and exhibited inconsistent myogenic potential across different preparations of MDSCs (Pisani et al., 2010b). When MDSCs were sorted into CD34^−^/CD90^+^, CD34^−^/CD90^−^ and CD34^+^/CD90^−^ fractions, CD34^+^/CD90^−^ cells showed the lowest expression of Myf5, MyoD, and Myogenin (Proksch et al., 2009). Consistently, in a different study using CD133^+^ myoblasts isolated from adult muscle, CD133^+^/CD34^−^ cells exhibited higher CD56 expression in culture compared to CD133^+^/CD34^+^ cells (Negroni et al., 2009). In another study, CD34^−^/CD56^+^/CD144^−^ cells also retained a high level of CD56 even after expansion in culture, whereas these cells also maintained a myogenic and endothelial cell phenotype (Okada et al., 2012). Interestingly, CD34^−^ MDSCs did not show adipogenic differentiation both *in vitro* and *in vivo*, whereas CD34^+^ cells were able to generate adipocytes both in culture and following transplantation (Pisani et al., 2010b). CD34^+^ MDSCs may retain cellular characteristics as multipotent stem cells, which possibly implies their limited commitment to myogenic lineage.

Although CD34^+^ cells do not specifically represent SMPC pools, these cells may have supporting roles for the integration of SMPCs when transplanted with CD34^+^ SMPCs. In one study, three cell populations (CD56^+^/CD34^+^/CD144^+^, CD56^−^/CD34^+^/CD144^+^, and CD56^+^/CD34^−^/CD144^−^) were isolated from human adult muscle as myoendothelial cells, endothelial cells, and myogenic cells, respectively. When their ability of cell survival and muscle regeneration was compared by intramuscular implantation into SCID mice, CD56^+^/CD34^+^/CD144^+^ myoendothelial cells demonstrated the most significant results (Zheng et al., 2007). In a different study, CD133^+^/CD34^+^ myoblasts isolated from human adult muscle showed better regeneration capacity compared to CD133^+^/CD34^−^ cells following transplantation into the tibialis anterior muscles of immunodeficient mice (Negroni et al., 2009).

#### CD45

CD45, also known as leukocyte common antigen or protein tyrosine phosphatase receptor type C, is a hematopoietic marker (Kaplan et al., 1990) widely used as a negative marker for SMPCs. In a number of studies, CD45^+^ cell depletion has already been used to enrich human SMPCs derived from adult muscle and fetal muscle (Zheng et al., 2007, 2012; Crisan et al., 2008; Lecourt et al., 2010; Okada et al., 2012; Bareja et al., 2014; Castiglioni et al., 2014; Xu et al., 2015; Garcia et al., 2018). In other studies, the absence of CD45 was used to define SMPC pools from the cell preparation derived from PSCs, adult muscle, placenta, or umbilical cord blood (Alessandri et al., 2004; Morosetti et al., 2006; Dellavalle et al., 2007; Nunes et al., 2007; Zheng et al., 2007; Proksch et al., 2009; Park et al., 2011; Awaya et al., 2012; Darabi et al., 2012; Tedesco et al., 2012; Castiglioni et al., 2014; Charville et al., 2015; Lorant et al., 2018).

#### CD57

CD57 is also known as beta-1,3-glucuronyltransferase 1 (B3GAT1), human natural killer 1 (HNK-1), or LEU-7. CD57 is a neuroectodermal marker expressed on natural killer cells and T-lymphocytes (Kared et al., 2016). Although knowledge about CD57 expression on SMPCs is relatively limited at this moment, a few studies reported that CD57 was useful to deplete neural cells during the purification of PSC-derived SMPCs (Borchin et al., 2013; Choi et al., 2016; Sakai-Takemura et al., 2018). When CD57 alone was used for sorting PSC-derived cells, only negative cells could differentiate into myotubes (Sakai-Takemura et al., 2018). Compared to CD56^−^ and CD57^+^/CD56^+^ cells, CD57^−^/CD56^+^ cells showed significantly increased expression of MyoD1, Myogenin, and MyHC. CD57^−^/CD56^+^ cells also showed higher expression of Myogenin and Pax7 than human fetal skeletal muscle and undifferentiated human ESCs (Choi et al., 2016). In a different study, CD57^−^/CD56^+^ PSC-derived SMPCs showed higher expression of Pax7, Myf5, and MyHC than unsorted cells in culture. However, there was no difference in engraftment rate between CD57^−^/CD56^+^ cells and unsorted cells when intramuscularly transplanted into mdx-NSG mice (Hicks et al., 2017).

#### CD106

CD106 (vascular cell adhesion molecule 1) has been known to show expression in MSCs, activated endothelial cells, and macrophages (Yang et al., 2013). Although murine satellite cells express CD106 (Liu et al., 2015; Maesner et al., 2016), it seems that CD106 expression in human SMPCs remains controversial. Some studies showed that the expression of this marker was positive in adult muscle-derived SMPC populations (Sinanan et al., 2004; Lecourt et al., 2010) while other studies reported an absence of CD106 expression in different types of SMPCs derived from PSCs, adult muscle and fetal muscle (Morosetti et al., 2006; Dellavalle et al., 2007; Crisan et al., 2008; Pisani et al., 2010b; Awaya et al., 2012; Darabi et al., 2012). Further studies would be required to determine the expression of CD106 on human SMPCs.

#### CD108

CD108, or semaphorin 7A, is a glycophosphatidylinositol-linked glycoprotein. When CD108 was used as a sole marker for sorting iPSC-derived SMPCs, CD108^−^ cells showed significantly higher levels of myotube formation *in vitro* compared to the unsorted cells. Isolated CD57^−^/CD108^−^/CD271^+^/ErbB3^+^ cells from iPSC-derived SMPCs were able to form myotubes with high efficiency in culture and when transplanted into the tibialis anterior muscles of immunodeficient dystrophin-deficient NSG-mdx^4Cv^ mice (Sakai-Takemura et al., 2018). In adult skeletal muscle, CD108 was expressed on both fibroblasts and myoblasts; this discrepancy is not well-understood (Sakai-Takemura et al., 2018).

### Combinations of Surface Markers Used for SMPC Enrichment

To date, cell sorting by several combinations of cell surface markers has worked successfully to enrich human PSC-derived SMPCs and to deplete undesirable cell types (Table 2). Although these individual works supported the feasibility of SMPC isolation using different combinations of surface markers, one common challenge is that the procedure becomes complicated with multiple sorting steps.

**Table 2:** Combinations of markers used to isolate human PSC-derived SMPCs and findings about their enrichment efficiency.

**Isolated fractions**	**PSCs**	***In vitro* analysis**	***In vivo* analysis**	**References**
CD10^+^/CD24^−^	Pax7/Myf5 dual reporter ESCs, and three transgene-free PSC lines	In all four cell lines, only CD10^+^/CD24^−^ cells exhibited myotube formation. CD10^+^/CD24^+^ CD10^−^/CD24^−^, and CD10^−^/CD24^+^ fractions contained non-myogenic cells and did not exhibit myotube formation	CD10^+^/CD24^−^ cells were sorted from Pax7/Myf5 dual reporter ESCs and transplanted into non-irradiated, cardiotoxin-damaged hindlimb (tibialis anterior, TA) muscles of immunodeficient dystrophin-deficient NSG-mdx^4Cv^ mice (2.5 × 10^5^ cells/10 μL/muscle). At 4 weeks post-transplantation, CD10^+^/CD24^−^ cells formed maximum of 50–60 dystrophin^+^ and lamin A/C^+^ fibers, where 12 ± 1.9% out of all lamin A/C^+^ cells were Pax7^+^. CD10^+^/CD24^−^ cells isolated from transgene-free PSCs were also able to form human myofibers in NSG-mdx^4Cv^ mice	Wu et al., 2018
CD57^−^/CD108^−^/CD271^+^/ ErbB3^+^	iPSCs	At 48 h after serial sorting, most cells were MyoD^+^, 30-40% were Pax7^+^. The cells formed Myogenin^+^ multinucleated myotubes. Pax7 was expressed in mononuclear cells between myotubes. When co-cultured with human adult myoblasts, the efficiency of myotube fusion was higher in the sorted cells compared to the unsorted ones	When the sorted cells were implanted into the TA muscles of NSG-mdx^4Cv^ mice, 12–13 myofibers with lamin A/C^+^ and Spectrin^+^ were found on transverse muscle sections at 3 weeks of post-transplantation. 1 × 10^5^ sorted cells formed more human lamin A/C^+^, spectrin^+^ and dystrophin^+^ myofibers compared to 1 × 10^6^ unsorted cells	Sakai-Takemura et al., 2018
CD54^+^/ α9β1^+^/ CD362^+^	PSCs with Pax7 overexpression by doxycycline-based induction	100% of cells were Pax7^+^. The isolated progenitors did not show expression of pluripotent stem cell markers (Oct3/4, Sox2 and Nanog) and were able to form multinucleated MyHC^+^ myotubes following terminal differentiation	Sorted cells were transplanted into cardiotoxin-injured TA muscles of NSG mice (5 × 10^5^ cells/10 μL/muscle). At 8 weeks and 10 months post-transplantation, ~50 and ~40 dystrophin^+^/lamin A/C^+^ myofibers were identified in each muscle section, respectively. Cell transplantation into NSG-mdx^4Cv^ mice showed similar results. Lamin A/C^+^ cells, which also expressed M-cadherin or Pax7, were detected in satellite cell position	Magli et al., 2017
CD56^+^/CD82^+^	iPSCs	When compared to double-negative cells, Pax7 and MyoD expression was found exclusively in CD56^+^/CD82^+^ cells. When compared to CD56^−^/CD82^+^, CD56^+^/CD82^−^ and double-negative cells, MyHC^+^ myotube formation was actively promoted in CD56^+^/CD82^+^ cells		Uezumi et al., 2016
CD57^−^/CD56^+^	PSCs	98% of single CD57^−^/CD56^+^ cells showed higher expression of Myogenin and Pax7 than human fetal skeletal muscle and undifferentiated ESCs. CD57^−^/CD56^+^ cells also expressed MyoD1, Myogenin and MyHC significantly higher than CD56^−^ cells and CD57^+^/CD56^+^ cells. Gene expression analysis revealed that key markers and transcription factors of skeletal muscle structure development were enriched in the CD57^−^/CD56^+^ cell fractions. CD57^−^/CD56^+^ cells could be expanded up to the hundreds of millions of cells and were easily cryo-preserved without losing myotube-forming competence	The CD57^−^/CD56^+^ cells were prepared from genetically corrected DMD iPSC-derived SMPCs and then transplanted into irradiated cardiotoxin-injured TA muscles of NSG-mdx^4Cv^ mice and immunodeficient NOD-Rag1^null^IL2rγ^null^ (NRG) mice (2 × 10^6^ cells/muscle). ~100 and ~380 laminin^+^/lamin A/C^+^ myofibers per muscle section were detected in NSG-mdx^4Cv^ mice and NRG mice, respectively	Choi et al., 2016
	PSCs	CD57^−^/CD56^+^ cells were positive with Pax7 and Myf5, specifically with ~1.7-fold more compared to unsorted preparations	Sorted cells were transplanted into cardiotoxin-injured TA muscles of NSG-mdx^4Cv^ mice (2 × 10^6^ cells/10 μL/muscle). CD57^−^/CD56^+^ cells did not show improved engraftment rate compared to unsorted cells. Significant differences were not found in spectrin^+^/lamin A/C^+^ and dystrophin^+^ myofibers generated by both groups of cells	Hicks et al., 2017
CD271^+^/ErbB3^+^	PSCs	CD271^+^/ErbB3^+^ expressed Pax7 and Myf5 20-fold more than double-negative cells. CD271^+^/ErbB3^+^ cells showed significantly higher myotube formation compared to CD271^+^/ErbB3^−^, CD271^−^/ErbB3^+^ and double-negative cells	DMD iPSC were genetically corrected by CRIPSR/Cas9 method and prepared SMPCs. CD271^+^ cells and ErbB3^+^ cells were sorted from these SMPCs and transplanted into cardiotoxin-injured TA muscles of NSG-mdx^4Cv^ mice (2 × 10^6^ cells/10 μL/muscle). Both cell types showed significantly higher engraftment rate than CD56^+^ and unsorted cells, as determined by the number of myofibers with human lamin A/C^+^/spectrin^+^/dystrophin^+^. Engraftment of ErbB3^+^ cells could restore dystrophin expression up to the levels observed in the muscles implanted freshly isolated human fetal myocytes	Hicks et al., 2017
	Pax7/Myf5 dual reporter ESCs, and three transgene-free PSC lines	At Day 5 and Day 15 in myogenic differentiation medium, CD271^+^/ErbB3^+^, CD271^+^/ErbB3^−^, and CD271^−^/ErbB3^+^ cells were evaluated with reporter protein expression (tdTomato driven by Pax7 promotor and EGFP by Myf5 promotor). At both timepoints, EGFP-positive myogenic cells were equally distributed in three fractions. When these fractions were plated and subjected to terminal differentiation, all of three fractions from Day 5 could not differentiate into myotubes. In contrast, all three fractions from Day 15 contained mixed populations of MyHC^+^ myotubes and MyHC^−^ non-myogenic cells	CD271^+^/ErbB3^+^ cells were isolated from Pax7/Myf5 dual reporter ESCs and transplanted into non-irradiated, cardiotoxin-damaged TA muscles of NSG-mdx^4Cv^ mice (2.5 × 10^5^ cells/10 μL/muscle). At 4 weeks post-transplantation, CD271^+^/ErbB3^+^ cells were able to form maximum of 10–15 dystrophin^+^ and lamin A/C^+^ myofibers per section, but no Pax7^+^ cells were detected	Wu et al., 2018
CD73^+^/CD56^+^	ESCs	60-80% of CD73^+^/CD56 cells were MyoD^+^. Cells adopted a bipolar cell morphology at 24 h in culture; 46 ± 3% and 7 ± 3% of total cells were MyoG^+^ and Pax7^+^, respectively. Upon terminal differentiation, the expression of Myogenin, desmin, actin and MyHC were confirmed. These differentiated myotubes were capable of spontaneous twitching in culture	Before transplantation, CD73^+^/CD56^+^-sorted cells were transduced with a lentiviral vector carrying a triple reporter construct expressing herpes simplex virus thymidine kinase, enhanced GFP, and luciferase. The cells were then transplanted into the cardiotoxin-injured TA muscles of immunodeficient SCID/Beige mice (5 × 10^5^ cells/muscle). Stable bioluminescence signals were confirmed even after 6 months of cell transplantation. Immunohistochemical analyses of implanted muscles demonstrated that an average of 7% of muscle fibers was positive with human laminin	Barberi et al., 2007
CD57^−^/AChR^−^/CD184^+^ /c-Met^+^	PSCs	Cell sorting was performed at three time points (Day 23, 25, and 35) following directed myogenic differentiation of PSCs. At Day 23, sorted cells expressed Six4 and Pax3. At Day 25, sorted cells expressed Pax7. At Day 35, sorted cells were 98% Pax3^+^ and 96% Pax7^+^. The cells sorted at Day 35 demonstrated progressive terminal muscle differentiation when plated down, as shown by expression of Myf5, Myogenin, and MyHC. After 3 days of terminal differentiation, all cells expressed MYF5 and few retained Pax7 expression. After 9 days, myotubes were formed while most cells were Myogenin^+^ and MyHC^+^		Borchin et al., 2013

### Which Surface Markers Would Be Promising for Human SMPC Isolation?

Based on our comprehensive search of the literature as described above, we summarize the promising surface markers that have already been used to enrich human PSC-derived SMPCs (Table 3; the rows of these markers are highlighted in gray). We include possible markers that have confirmed expression in human PSC-derived SMPCs but have not been tested yet for sorting human PSC-derived SMPCs (Table 3; the rows of these markers remain white). We also made note of whether they were used alone or in combination with other markers, and in which PSC lines the markers have been tested. Although this is not a definitive list, we consider the bolded markers in Table 3 to be highly promising for the enrichment of human PSC-derived SMPCs: CD10, CD54, CD56, CD82, CD271, ErbB3, and c-Met for positive selection; and CD24, CD57 for negative selection. Although it is difficult to propose the best combination of these markers at this moment, several combinations have successfully worked as described in the previous studies. They include CD10^+^/CD24^−^ (Wu et al., 2018), CD57^−^/CD108^−^/CD271^+^/ErbB3^+^ (Sakai-Takemura et al., 2018), CD56^+^/CD82^+^ (Uezumi et al., 2016), CD271^+^/ErbB3^+^ (Hicks et al., 2017), CD57^−^/CD56^+^ (Choi et al., 2016; Hicks et al., 2017), and CD57^−^/AChR^−^/CD184^+^/c-Met^+^ (Borchin et al., 2013; Table 2). To further improve the efficiency of SMPC enrichment, additional studies would be necessary to test different combinations.

**Table 3:** Surface markers and their potential to isolate human PSC-derived SMPCs.

**Marker**	**+/– selection**	**Strength**	**Weakness**	**Expression level in SMPCs from other sources**
**CD10**	**+**	• Enriched SMPC population from transgene-free PSCs and Pax7/Myf5 reporter ESCs when used in combination with CD24^-^ selection (Wu et al., 2018)	• Efficiency as a sole marker is unknown • Efficiency when used with markers other than CD24^−^ is unknown	• Detected on ~97% human primary myoblasts (Wu et al., 2018) • Detected on ~80% Pax7^+^ cells derived from adult muscle (Wu et al., 2018)
CD11b	**-**	• Exclusion of CD11b^+^ cells efficiently depleted hematopoietic cells among SMPC population derived from fetal muscle, when used in combination with GlyA^-^ /CD45^-^ selection (Castiglioni et al., 2014), and from adult muscle when used in combination with CD45^-^ selection (Bareja et al., 2014)	• May be redundant as a negative marker, as the expression on human PSC-derived SMPCs was rarely reported • CD11b^+^ population from CRISPR/Cas9 corrected DMD iPSC-derived SMPC was able to form myotube in culture (Hicks et al., 2017)	
CD13	**+**	• Expression was detected on 40-64% human Pax7-overexpressing PSC-derived SMPCs (Darabi et al., 2012) and iPSC-derived mesoangioblast-like stem/progenitor cells (Tedesco et al., 2012)	• Never used to isolate human PSC-derived SMPCs, efficiency is unknown whether as a sole marker or in combination	• Detected on ~97% human adult pericytes (Dellavalle et al., 2007) • Detected on 38.4% mononucleated cells derived from fetal muscle (Castiglioni et al., 2014)
CD15	**-**	• Presence or absence of expression could distinguish CD56^+^ adult muscle-derived myogenic cells with or without adipogenic capabilities, respectively (Lecourt et al., 2010; Pisani et al., 2010a; Agley et al., 2013)	• May be redundant as a negative marker, as CD15 expression on human PSC-derived SMPCs has not been confirmed yet	
**CD24**	−	• Enriched SMPC population from transgene-free PSCs and Pax7/Myf5 reporter ESCs when used in combination with CD10^+^ selection (Wu et al., 2018)	• Efficiency as a sole marker is unknown • Efficiency when used with markers other than CD10^+^ is unknown	• Not detected on human primary myoblasts (Wu et al., 2018)
CD29	**+**	• CD29/Integrin alpha-9 dimer (α9β1) efficiently isolated SMPC population from Pax7-overexpressing PSCs (Magli et al., 2017) • Expression confirmed by >70% cells of PSC-derived SMPC populations (Awaya et al., 2012; Darabi et al., 2012; Abujarour et al., 2014; Magli et al., 2017; Sakai-Takemura et al., 2018) • Enriched SMPC population from adult muscle when used in combination with CD31^−^/CD34^−^/CD45^−^/EGFR^+^ selection (Charville et al., 2015), and with CD56^+^/CD31^−^/CD45^−^ selection (Xu et al., 2015), as well as from fetal muscle when used in combination with CD56^+^/CD184^+^/CD31^−^/CD34^−^/CD45^−^ selection (Garcia et al., 2018)	• Never used alone (as opposed to α9β1 dimer) to isolate human PSC-derived SMPCs; efficiency is unknown whether as a sole marker or in combination • A population of CD56^+^/CD29^+^ cells without Pax7 expression was identified in healthy adult human skeletal muscle, implying that CD29 expression is not exclusive to satellite cells (Xu et al., 2015)	• Detected on 90.4% mononucleated cells derived from fetal muscle (Castiglioni et al., 2014) • Co-expression with CD56 was detected on 100% Pax7^+^ satellite cells in healthy adult skeletal muscle (Xu et al., 2015) • Detected on SMPCs derived from postnatal muscle (Garcia et al., 2018) and adult muscle (Lecourt et al., 2010; Woodard et al., 2014; Charville et al., 2015; Xu et al., 2015; Lorant et al., 2018)
CD31	**-**	• Exclusion of CD31^+^ cells in a series of sequential isolation steps efficiently depleted endothelial cells among SMPC population derived from adult muscle (Bareja et al., 2014; Xu et al., 2015; Garcia et al., 2018) and fetal muscle (Cerletti et al., 2006; Castiglioni et al., 2014)	• May be redundant as a negative marker for PSC-derived SMPCs as expression was not detected on human Pax7-overexpressing PSC-derived SMPCs (Darabi et al., 2012) and iPSC-derived mesoangioblast-like stem/progenitor cells (Tedesco et al., 2012) • CD31 expression itself does not correspond to SMPC properties, as both CD146^+^/CD31^+^ and CD146^+^/CD31^−^ cells isolated from human fetal muscle and cultured were able to differentiate into myotubes (Cerletti et al., 2006)	• Expression on SMPCs derived from adult muscle had been reported to be both detectable (Hollemann et al., 2008; Lecourt et al., 2010; Bareja et al., 2014; Xu et al., 2015) and undetectable (Alessandri et al., 2004; Dellavalle et al., 2007; Charville et al., 2015; Lorant et al., 2018)
CD34	**-**	• CD34^−^ MDSCs displayed high expression of myogenic markers and exhibited consistent myogenic potential regardless of preparation and culture period (Pisani et al., 2010b) • CD34^−^ MDSCs retained higher expression of CD56 in culture when sorted along with both CD90^+^ and CD90^−^ selections (Proksch et al., 2009), CD133^+^ selection (Negroni et al., 2009), as well as CD56^+^/CD144^−^ selection (Okada et al., 2012)	• May be redundant as a negative marker for PSC-derived SMPCs as expression was reported to be non-existent or low on human PSC-derived SMPCs (Awaya et al., 2012; Darabi et al., 2012; Hwang et al., 2014)	• Some groups of SMPCs have been confirmed as CD34^−^ populations: proliferative satellite cells (CD34^−^/CD56^+^/Myf5^+^ cells), and a minority of pericytes and mesoangioblasts with adipogenic potential (CD34^−^/CD56^−^ cells) (Péault et al., 2007). • Expression reported to be in varied levels on SMPCs from different sources (see section CD34)
CD45	**-**	• Exclusion of CD45^+^ cells in a series of sequential isolation steps efficiently depleted hematopoietic cells among SMPC population derived from adult muscle (Zheng et al., 2007, 2012; Lecourt et al., 2010; Okada et al., 2012; Bareja et al., 2014; Xu et al., 2015) and fetal muscle (Crisan et al., 2008; Castiglioni et al., 2014; Garcia et al., 2018)	• May be redundant as a negative marker for PSC-derived SMPCs as expression was not detected on human Pax7-overexpressing PSC-derived SMPCs (Darabi et al., 2012) and iPSC-derived mesoangioblast-like stem/progenitor cells (Tedesco et al., 2012)	• Not detected on SMPC populations derived from adult muscle, placenta, and umbilical cord blood (see section CD45)
**CD54**	**+**	• Enriched SMPC population from Pax7-overexpressing PSCs when used alongside with CD362^+^/integrin α9β1^+^ selection (Magli et al., 2017) • Able to act as a sole marker to isolate SMPC population from Pax7-overexpressing PSCs (Magli et al., 2017)	• Efficiency to isolate transgene-free PSC-derived SMPCs is unknown	• Expression on cultured human skeletal muscle cells can be induced with various cytokine treatments (Goebels et al., 1992; Michaelis et al., 1993; Bartoccioni et al., 1994; Marino et al., 2001)
**CD56**	**+**	• Enriched SMPCs from transgene-free ESC-derived MSCs when used alongside CD73^+^ selection (Barberi et al., 2007) • Enriched transgene-free iPSC-derived SMPCs when used alongside CD82^+^ selection (Uezumi et al., 2016) • Enriched transgene-free PSC-derived SMPCs when used alongside CD57^−^ selection (Hicks et al., 2017) • Enriched SMPC population derived from fetal muscle (Crisan et al., 2008; Castiglioni et al., 2014) and adult muscle (Dellavalle et al., 2007; Zheng et al., 2007, 2012; Lecourt et al., 2010; Pisani et al., 2010a; Okada et al., 2012; Bareja et al., 2014; Woodard et al., 2014; Xu et al., 2015; Alexander et al., 2016; Choi et al., 2016) alongside other markers • Able to act as a sole marker to isolate SMPC population derived from adult muscle (Sinanan et al., 2004; Lecourt et al., 2010; Pisani et al., 2010a; Lorant et al., 2018)	• Efficiency to isolate transgene-based PSC-derived SMPCs is unknown • Efficiency as a sole marker to isolate PSC-derived SMPCs is unknown • May not improve SMPC purity by much as almost all cells in some PSC-derived SMPC populations express CD56: 98-100% of human Pax7-overexpressing PSC-derived SMPCs (Darabi et al., 2012), 95.3% of Pax3^+^ or Pax7^+^ cells from ESC-derived SMPCs (Awaya et al., 2012), 75% MyoD-overexpressing ESCs (Goudenege et al., 2012) • SMPCs may also reside in CD56^−^ population, as evidenced by several CD56^−^ populations that were able to form myotubes both *in vitro* and *in vivo*: a population of “endothelial cells” (CD45^−^/CD56^−^/CD34^+^/CD144^+^ selection) isolated from adult muscle (Okada et al., 2012), a population of “perivascular stem cells” (CD146^+^/CD45^−^/CD56^−^/UEA-1R^−^ selection) derived from adult muscle (Zheng et al., 2012), and a population of “perivascular cells” (CD146^+^/CD34^−^/CD45^−^/CD56^−^ selection) isolated from multiple human organs and placenta (Crisan et al., 2008; Park et al., 2011); the latter two populations showed upregulated CD56 expression after expression in culture	• Detected on >80% satellite cells derived from adult muscle (Negroni et al., 2009) • Detected on 83% “slowly adhering cell” fraction of culture derived from adult muscle (Okada et al., 2012) • Detected on 77.1% CD45^−^ cultured cells derived from adult muscle (Zheng et al., 2012) • Detected on 69-99% SMPCs derived from adult muscle (Lorant et al., 2018) • Detected on SMPCs derived from PSCs, postnatal muscle, adult muscle, fetal muscle, and placenta (see section CD56)
**CD57**	−	• Exclusion of CD57^+^ cells in a series of sequential isolation steps efficiently depleted neural cells among transgene-free PSC-derived SMPC populations (Borchin et al., 2013; Hicks et al., 2017; Sakai-Takemura et al., 2018) and Mesogenin1/eGFP reporter human ESC-derived SMPC population (Choi et al., 2016) • Able to enrich transgene-free iPSC-derived SMPCs when used as a sole marker in a single-step negative selection (Sakai-Takemura et al., 2018)	• May be redundant as a negative marker as expression on human PSC-derived SMPCs was rarely reported • May not improve SMPC purity by much if expression level is low in PSC-derived SMPC populations	
CD73	**+**	• The expression was confirmed in 99.4% Pax3^+^ or Pax7^+^ cells from ESC-derived SMPCs (Awaya et al., 2012), 96.8% MyoD-overexpressing ESCs (Goudenege et al., 2012) and 66% SMPCs derived from adult muscle (Woodard et al., 2014)	• Never used to isolate SMPCs from any source, efficiency is unknown whether as a sole marker or in combination	• Detected on SMPCs derived from fetal muscle (Crisan et al., 2008) and adult muscle (Lecourt et al., 2010; Uezumi et al., 2016; Lorant et al., 2018)
**CD82**	**+**	• Enriched transgene-free iPSC-derived SMPCs (Uezumi et al., 2016) and SMPCs derived from adult muscle (Alexander et al., 2016) when used alongside CD56+ selection • Enriched SMPCs derived from fetal muscle when used alongside CD146^+^ selection (Alexander et al., 2016) • Can be used alone to enrich SMPCs derived from adult muscle (Uezumi et al., 2016)	• Efficiency to isolate transgene-based PSC-derived SMPCs is unknown • Efficiency as a sole marker to isolate PSC-derived SMPCs is unknown • Fairly new marker, expression level in PSC-derived SMPCs have not been reported much, therefore hard to gauge redundancy of isolation based on this marker	• Detected on ~97% Pax7^+^ or M-cadherin^+^ satellite cells (Uezumi et al., 2016)
CD108	−	• Enriched transgene-free iPSC-derived SMPCs as a sole marker and when used alongside CD57^−^/CD271^+^/ErbB3^+^ selection (Sakai-Takemura et al., 2018)	• Efficiency to isolate transgene-based PSC-derived SMPCs is unknown • Efficiency as a sole marker or when used with markers other than CD57^−^/CD271^+^/ErbB3^+^ is unknown • Expression level in PSC-derived SMPCs is rarely reported, therefore hard to gauge redundancy of isolation based on this marker	• Detected on both fibroblasts and myoblasts in adult skeletal muscle (Sakai-Takemura et al., 2018)
CD146	**+**	• Expression confirmed in PSC-derived SMPCs (Darabi et al., 2012; Tedesco et al., 2012; Sakai-Takemura et al., 2018) • Enriched SMPC population derived from fetal muscle (Cerletti et al., 2006; Crisan et al., 2008; Lapan et al., 2012; Alexander et al., 2016), adult muscle (Zheng et al., 2012) and placenta (Park et al., 2011)	• Efficiency to isolate PSC-derived SMPCs is unknown • May not improve SMPC purity by much if very highly expressed in PSC-derived SMPC populations; for example, 100% of human Pax7-overexpressing PSC-derived SMPCs were CD146^+^ (Darabi et al., 2012)	• Detected on 41.11% culture-expanded pericytes derived from adult muscle (Dellavalle et al., 2007) • Detected on 66.9% CD45^−^ cultured cells derived from adult muscle (Zheng et al., 2012) • Detected on 35-80% mononucleated cells derived from fetal muscle (Lapan et al., 2012)
CD184	**+**	• Enriched highly pure SMPCs from transgene-free PSC-derived populations when used in combination with CD57^−^/AChR^−^/c-Met^+^ selection (Borchin et al., 2013)	• Does not exclusively select SMPCs. Also expressed on neural cells, therefore most likely only reliable when used alongside c-Met: highly pure SMPCs were found to reside in c-Met^+^/CD184^−^ population; c-Met^−^/CD184^+^ fraction contained both SMPCs and neural cells; c-Met^−^/CD184^−^ fraction did not have any SMPCs (Borchin et al., 2013) • Efficiency when used with markers other than c-Met is unknown	• Detected on 63.2% mononucleated cells derived from fetal muscle (Castiglioni et al., 2014) • Detected on SMPCs derived from adult muscle (Bareja et al., 2014; Marg et al., 2014; Garcia et al., 2018)
**CD271**	**+**	• Enriched transgene-free iPSC-derived SMPC population when used as a sole marker and when used in combination with CD57^−^/CD108^−^/ErbB3^+^ selection (Sakai-Takemura et al., 2018) • Resulted in the most efficient SMPC enrichment from CRISPR/Cas9 corrected DMD iPSC-derived SMPC population when used as a sole marker, compared to CD11b, CD56, CD146, CD184, c-Met, ITGA7, ErbB3 used as sole markers (Hicks et al., 2017) • Enriched transgene-free and transgene-based PSC-derived SMPC populations when used alongside ErbB3^+^ selection (Hicks et al., 2017)	• May not be able to isolate SMPCs in all human PSC-derived SMPC populations; failed to enrich for SMPCs in a Pax7/Myf5 reporter ESC population when used alongside ErbB3^+^ or ErbB3^−^ selection (Wu et al., 2018) • Fairly new marker; expression level in PSC-derived SMPCs has not been reported much, therefore hard to gauge redundancy of isolation based on this marker	• Detected on cultured-expanded postnatal myoblasts (Sakai-Takemura et al., 2018) • Detected in a subpopulation co-expressing CD271^+^ and myogenic transcription factors in the muscle at 8 weeks of gestation; these cells then started losing CD271 expression at 16 weeks of gestation
CD318	**+**	• Expression reported in 43.1% of transgene-free iPSC-derived SMPCs (Sakai-Takemura et al., 2018)	• Failed as a sole marker to enrich transgene-free iPSC-derived SMPCs due to undetectable expression level (Uezumi et al., 2016) • Efficiency to isolate PSC-derived SMPCs in combination with other markers is unknown	• Cytoplasmic expression was detected in ~75% of satellite cells (Uezumi et al., 2016) • Only the CD318+ fraction contained Pax7^+^/MyoD1^+^ adult muscle-derived SMPCs grown in hypoxic conditions (Alexander et al., 2016)
CD362	**+**	• Enriched SMPC population from Pax7-overexpressing PSCs when used alongside with CD54^+^/integrin α9β1^+^ selection (Magli et al., 2017) • Detected on ~95% CD54^+^/integrin α9β1^+^ proliferating SMPCs	• Efficiency to isolate transgene-free PSC-derived SMPCs is unknown • Efficiency as a sole marker or when used with markers other than CD54^+^/integrin α9β1^+^ is unknown • Expression level in PSC-derived SMPCs is rarely reported, therefore hard to gauge redundancy of isolation based on this marker	
**c-Met**	**+**	• Enriched highly pure SMPCs and depleted neural cells and other non-myogenic cell types from transgene-free PSC-derived populations when used in combination with CD57^−^/AChR^−^ selection (Borchin et al., 2013)	• May be redundant if not expressed on human PSC-derived SMPCs; expressed only in 2.4% cells of an iPSC-derived SMPC population (Sakai-Takemura et al., 2018); failed to act as a sole marker to enrich CRISPR/Cas9 corrected DMD iPSC-derived SMPCs (Hicks et al., 2017) • Efficiency as a sole marker is unknown	
**ErbB3**	**+**	• When used as a sole marker yielded better SMPC enrichment from transgene-free iPSC-derived SMPC population than CD56^+^/CD82^+^ selection (Sakai-Takemura et al., 2018) • Enriched transgene-free iPSC-derived SMPC population when used in combination with CD57^−^/CD108^−^/ErbB3^+^ selection (Sakai-Takemura et al., 2018) • Resulted in the second most efficient SMPC enrichment from CRISPR/Cas9 corrected DMD iPSC-derived SMPC population when used as a sole marker, compared to CD11b, CD56, CD146, CD184, CD271, c-Met, ITGA7 used as sole markers (Hicks et al., 2017)	• May not be able to isolate SMPCs in all human PSC-derived SMPC populations; failed to enrich for SMPCs in a Pax7/Myf5 reporter ESC population when used alongside CD271^+^ or CD271^−^ selection (Wu et al., 2018) • Fairly new marker; expression level in PSC-derived SMPCs have not been reported much, therefore hard to gauge redundancy of isolation based on this marker	• Detected on CD146^+^ SMPCs derived from fetal muscle (Alexander et al., 2016) • Detected in a subpopulation co-expressing ErbB3^+^ and myogenic transcription factors in the muscle at 8 weeks of gestation; these cells at 16 weeks of gestation exhibited close to 100% myotube-forming capability if plated down

*Markers with gray background have already been used to isolate human PSC-derived SMPCs. The markers with white background are the ones that have not been tested for sorting of PSC-derived SMPC but are potentially useful for SMPC isolation. Based on our literature search, we propose that the bolded markers would be highly promising for SMPC isolation from human pluripotent sources*.

## Remaining Challenges to Isolate Human SMPCs From Pluripotent Stem Cells

The current knowledge of cell surface markers supports our ability to isolate and characterize PSC-derived SMPCs. While a number of positive and negative markers are potentially available to define SMPC pools, it may still not be easy to prepare a pure population of SMPCs from PSC-derived cells.

In recent years, several culture protocols have been developed to derive skeletal myocytes from human PSCs. Transgene-based SMPC derivation uses the overexpression of myogenic genes, such as PAX7 (Darabi et al., 2012; Skoglund et al., 2014) and MYOD1 (Tanaka et al., 2013; Abujarour et al., 2014; Yasuno et al., 2014; Maffioletti et al., 2015). Although transgene-based approaches yield SMPCs with high efficiency, the resulting cells may not fully reflect the natural endogenous processes of SMPC proliferation, differentiation, and maturation, because such overexpression requires genetic modification (reviewed in Jiwlawat et al., 2018). Similarly, although fluorophore-labeled SMPCs warrant convenient isolation, the insertion of transgene constructs that link fluorophore reporter genes to myogenic genes present the same concerns associated with transgene-based approaches. As such, transgene-free methods may be more suitable to prepare SMPCs for clinical applications, because these methods only use defined culture conditions supplemented with factors that encourage myogenic lineage commitment. However, the efficiency of SMPC derivation is relatively lower in the existing transgene-free methods compared to transgene-based approaches. Further, transgene-free SMPC derivation gives rise to a heterogeneous cell population with an embryonic or perinatal phenotype which contains myocytes of various stages and other cell types (Jiwlawat et al., 2017). As such, to obtain SMPC populations with high quality and quantity, cell sorting using cell surface markers would be required. A better understanding of SMPC surface markers is crucial to improve the purity of resulting cell populations.

With the use of specific antibodies targeting only cells expressing a particular surface marker, positive selection yields a higher purity of the desired population. However, positively selected cells may retain antibodies and other labeling agents, which may interfere with downstream culture and assays. Negatively selected cells would not carry such concern, but it is less efficient in terms of purity to deplete all undesired cells by only relying on negative selection. Positive selection and negative selection can be used to purify a cell population sequentially through several cycles of the procedure.

When designing a strategy of SMPC enrichment, we should consider the expression level of each surface marker in unsorted populations. Intuitively, CD29 and CD56 are well-established surface markers that have been used for SMPC enrichment in various cell lines. However, if an SMPC surface marker is already very highly expressed in pre-sorted populations (>90%), it may be redundant to enrich using this surface marker, as the purity may not improve by much. The same applies to negative SMPC surface markers that are already minimally expressed in unsorted populations. A surface marker barely expressed in the unsorted population may play an important biological role but would not be useful as a target for live cell isolation. Similarly, if a surface marker is also highly expressed on non-SMPC cell types, it would no longer serve to distinguish SMPC identity from the background cell populations.

We also need to carefully compare the rate of improvement in muscle differentiation efficiency between the unsorted and sorted populations. When quantifying the success rate of SMPC enrichment, the *in vivo* capacity of muscle regeneration should be taken into consideration alongside the ability of the cells to differentiate into muscle *in vitro*. Forming new muscle fibers, repopulating the satellite cell niche, and vascularization of the affected area are all crucial to establish the structural integrity and functionality of the regenerated muscle tissue. It is possible that unsorted SMPC populations may perform better after transplantation, as these more heterogenous unsorted populations could contain cells that play a supporting role for muscle repair and regeneration. “Enriched” SMPC populations may show an increased rate of myotube formation *in vitro* where conditions are strictly controlled. However, these cells may engraft more poorly than unsorted populations due to the absence of non-SMPC support cells. Therefore, the purpose of SMPC enrichment should be considered when selecting surface markers, as the resulting cell population most ideal for *in vitro* modeling may not also be the most ideal for transplantation studies.

Our literature search further verifies that a surface marker profile cannot be assumed to be similar for all SMPC lines. A surface marker could enrich SMPCs from one cell line very well only to be rendered useless in another cell line. For example, CD271 and ErbB3 efficiently enriched SMPCs derived via both transgene-free and transgene-based methods from several PSC lines (Hicks et al., 2017; Sakai-Takemura et al., 2018). However in a different paper, when a combination of CD271 and Erb3 was used for an ESC line (Pax7/Myf5 reporter ESC), the similar efficiency of SMPC enrichment could not be replicated (Wu et al., 2018). Thus, more comprehensive isolation strategies that account for expression-level variability are warranted. The variance in antibody binding affinity from different suppliers should also be carefully considered when designing an antibody-based isolation strategy as well as in data interpretation.

When differentiating skeletal myocytes from human PSCs, there has been inconsistency between studies with regards to evaluating differentiation efficiency and myocyte maturity (reviewed in Jiwlawat et al., 2018). It would be greatly beneficial in the field to establish standards for these evaluations in order to make better comparisons across protocols. To achieve this goal, cell characterization using specific surface markers against PSC-derived SMPCs would be crucial. Another challenge in the field is that cultured skeletal myocytes often retain an embryonic or perinatal phenotype. While better establishment of culture technique would be required to obtain SMPCs and myocytes with sufficient quality, it would also be worthwhile to define stage-specific markers to distinguish embryonic, perinatal, and adult phenotypes of SMPCs and myoblasts.

To date, a majority of SMPC surface markers were profiled based on the knowledge from satellite cells, resident SMPCs located between the basal lamina and sarcolemma of adult muscle fibers. Even though these possible markers can be used to sort SMPCs into subpopulations, it remains uncertain whether these subpopulations are homogeneous in their function and lineage commitment. Moreover, given the fact that satellite cells from different *in situ* locations within the same donor can have distinct molecular signatures (Harel et al., 2009; Sambasivan et al., 2009), these markers should not be utilized alone to unequivocally identify SMPCs, especially “*de novo* SMPCs” derived from PSCs. Additionally, contradictory reports on marker expression on SMPCs imply that different culture conditions may lead to the heterogeneity of SMPCs. Different culture media and durations would influence the expression of specific markers in timepoint- and sample-dependent manners. Ultimately, it would be more helpful if a sole marker was available to specifically enrich only SMPCs. For other stem cell types such as HSCs and MSCs, cell surface markers are already well-characterized and defined for identification. In contrast, our knowledge in the field of myogenic progenitors is still developing. To advance, additional studies are needed to discover specific markers that can be used alone to detect a pure population of SMPCs.

Lastly, another concern is that throughout the reports there is inconsistency on how to evaluate the myogenic potential in sorted cell pools. Some studies examined commitment to the myogenic lineage based on a pooled expression of common muscle cell markers, whereas several other studies only identified the expression of one marker. When comparing *in vivo* regenerative capacity using intramuscular transplantation, the results may also vary depending on the animal models used, how muscle injury was induced, the number of cells transplanted, the duration between transplantation and analysis, and the position in muscle used for section preparation and analysis. Furthermore, calculation of myotube formation efficiency may differ based on the usage of immunohistochemical staining, the counting of positive cells in a field of view, the number of nuclei per myotube, and the percentage of nuclei within myotubes. Since the ability to generate fully matured myotubes is pivotal for transplanted SMPCs to have clinical significance, a comprehensive analysis of the anatomical features, physiological functionality and fiber type expressed should be performed to gauge the state of myotube maturity.

## Concluding Remarks

Recent studies have offered valuable knowledge regarding cell surface markers to identify SMPCs. While promising positive and negative markers have been identified using different SMPC types, it remains challenging to use them for sorting human PSC-derived SMPCs in efficient ways. Furthermore, there is still a need to standardize methods of quantifying SMPC properties in order to facilitate comparisons between surface marker expressions. This would allow for precise utilization of SMPC surface markers to enhance the enrichment and differentiation of SMPCs. Having control over the composition of SMPC populations could lead to new state of the art uses for disease modeling, drug testing, and therapeutic development.

## Author Contributions

S-RT designed the outline of manuscript, performed manuscript search, and primarily prepared the manuscript. SR and EL provided critical suggestions about the contents and edited the manuscript. MS proposed conception and design of the manuscript and wrote the manuscript. All authors approved the final version of the manuscript.

### Conflict of Interest

The authors declare that the research was conducted in the absence of any commercial or financial relationships that could be construed as a potential conflict of interest.
